# Melamine-Modified MIL-101(Cr) with Enhanced CO_2_ Affinity for Low-Pressure Capture

**DOI:** 10.1021/acsomega.6c07336

**Published:** 2026-09-15

**Authors:** Andrés Lancheros, Claudio Contreras-Díaz, Esteban Camú, Thiago C. dos Santos, Célia M. Ronconi, Néstor Escalona, Élodie Blanco

**Affiliations:** † Department of Chemical Engineering and Bioprocesses. Pontificia Universidad Católica de Chile, Santiago 7820436, Chile; ‡ Chemistry Institute, Universidade Federal do Rio de Janeiro, Rio de Janeiro, Rio de Janeiro 21941-901, Brazil; § Department of Inorganic Chemistry, Universidade Federal Fluminense, Niterói, Rio de Janeiro 24220-900, Brazil; ∥ Department of Engineering and Construction Management, Pontificia Universidad Católica de Chile, Santiago 7820436, Chile

## Abstract

MIL-101­(Cr) was post-synthetically modified with melamine to investigate the effect of nitrogen-rich functionalities on low-pressure CO_2_ adsorption behavior. Melamine incorporation partially reduced the accessible surface area and pore volume of the framework while preserving its crystallinity and structural stability. Despite the decrease in porosity, the modified MIL-101­(Cr)-MEL exhibited enhanced CO_2_ adsorption performance at low pressures and elevated temperatures compared with pristine MIL-101­(Cr). At 60 °C and 1 bar, the CO_2_ uptake of MIL-101­(Cr)-MEL was more than twice that of the pristine material, while the working capacity in the 0.08–0.14 bar range increased fourfold. IAST calculations revealed significantly enhanced CO_2_/N_2_ selectivity at low pressures, reaching selectivity values close to 90 below 0.05 bar. Dynamic breakthrough experiments under both dry and humid CO_2_/N_2_ gas streams further confirmed the preferential CO_2_ adsorption of MIL-101­(Cr)-MEL, with only a 13.4% reduction in CO_2_ adsorption capacity under humid conditions. The modified material also exhibited higher isosteric enthalpies of adsorption at low CO_2_ coverage, indicating enhanced CO_2_ affinity relative to pristine MIL-101­(Cr). However, the moderate −ΔH_ads_ values suggest that adsorption is governed by reversible non-covalent CO_2_-melamine interactions rather than by the formation of strongly bound CO_2_-amine adducts, providing an effective balance between CO_2_ affinity and regenerability. DRIFTS analyses confirmed reversible CO_2_ adsorption without evidence of carbonate or carbamate formation, supporting a mechanism dominated by moderate-strength non-covalent interactions. Overall, these results demonstrate that melamine functionalization provides a simple and effective strategy to enhance low-pressure CO_2_ capture performance under thermally demanding conditions.

## Graphical abstract

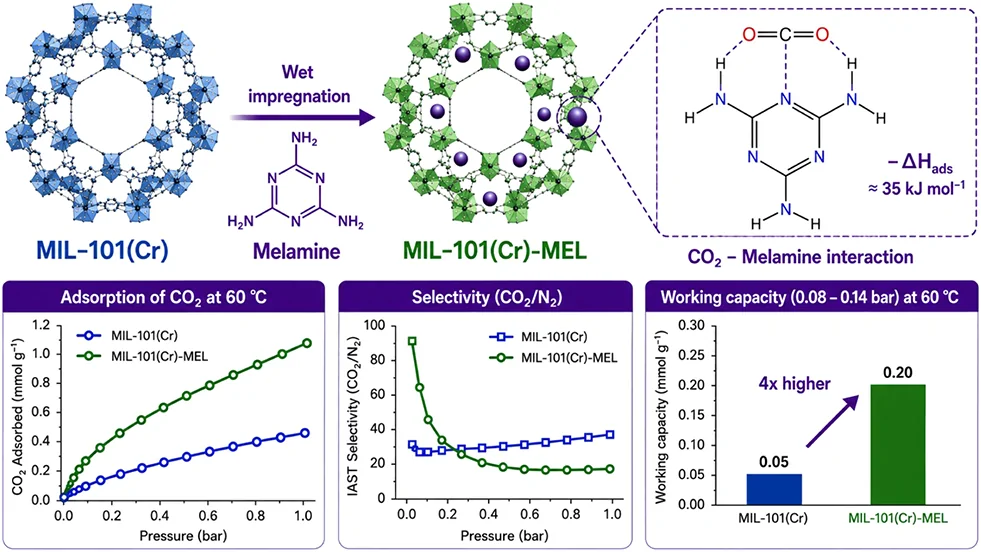

## Introduction

The continuous increase in atmospheric CO_2_ concentration caused by fossil fuel combustion remains one of the major environmental challenges associated with climate change and global warming.^1,2^ Among the different mitigation strategies, adsorption-based carbon capture technologies have attracted considerable attention because solid adsorbents can provide lower regeneration energy penalties and greater cyclic stability than conventional aqueous amine absorption systems.^3,4^ In particular, the capture of diluted CO_2_ streams under post-combustion and direct-air-capture-relevant conditions requires adsorbents capable of maintaining strong CO_2_ affinity at low partial pressures and elevated temperatures.^5,6^

Metal-organic frameworks (MOFs) have emerged as promising adsorbents for CO_2_ capture because of their exceptionally high surface areas, tunable pore structures, and versatile surface chemistry.^7–9^ Their structural diversity enables the rational design of porous environments with tailored adsorption properties, making them attractive materials for selective gas separation processes.^10–12^ However, under diluted CO_2_ conditions, adsorption performance becomes increasingly governed by the strength and density of specific adsorption sites rather than solely by the total accessible surface area or pore volume.^13^ Consequently, the incorporation of high-affinity adsorption sites capable of selectively interacting with CO_2_ molecules at low pressures has become an important strategy for improving the performance of porous adsorbents.^5,14^

Among the different MOFs investigated for CO_2_ capture, MIL-101­(Cr) has attracted particular interest because of its exceptional thermal and chemical stability, large pore volume, and high surface area, which facilitate gas diffusion and adsorption.^15–17^ Recent studies have further expanded the potential of MIL-101­(Cr) for acid gas capture through advanced structural engineering. Strategies including the incorporation of nitrogen-doped carbon quantum dots and graphene oxide nanocomposites,^18,19^ as well as the development of core-shell MIL-101­(Cr)@UiO-66­(Zr) heterostructures,^20^ have demonstrated enhanced CO_2_ and H_2_S adsorption and improved gas separation performance by tailoring pore architecture and increasing the availability of high-affinity adsorption sites.

Nevertheless, despite its highly porous structure, pristine MIL-101­(Cr) exhibits relatively low affinity toward CO_2_ at low pressures because its adsorption behavior is mainly dominated by weak physisorptive interactions.^16,21^ Therefore, several post-synthetic modification strategies have been explored to introduce nitrogen-containing functionalities capable of strengthening CO_2_-framework interactions and enhancing adsorption selectivity under diluted CO_2_ conditions.^14,22^

Amine functionalization is one of the most widely explored approaches to improve CO_2_ adsorption performance in porous materials due to the strong interactions that nitrogen-containing groups can establish with CO_2_ molecules.^23–25^ In many cases, these modifications increase CO_2_ uptake and selectivity by introducing stronger adsorption sites within the porous structure.^14^

However, conventional amine-functionalized systems also present several limitations that may compromise their long-term practical application. High amine loadings often lead to a significant reduction in the accessible surface area and pore volume of the porous support,^26^ resulting in diffusion limitations, slower adsorption-desorption kinetics, and higher regeneration energy requirements.^24,27^ In addition, amines may undergo oxidative degradation, volatilization, or leaching under operating conditions, while prolonged exposure to CO_2_ can promote irreversible urea formation, contributing to adsorbent deactivation.^28^ Furthermore, the presence of humidity and acid gases such as SO_2_ and NO_*x*_ can adversely affect adsorption performance through the formation of strongly bound sulfate and nitrate species that block active sites.^29^ Therefore, the development of adsorbents capable of providing moderate-strength, reversible CO_2_ interactions while preserving fast adsorption behavior and structural stability remains highly desirable for cyclic carbon capture applications.^30^

In this context, melamine (2,4,6-triamino-1,3,5-triazine) emerges as an attractive modifier because of its high nitrogen content, multiple amino functionalities, and ability to establish moderate-strength non-covalent interactions with CO_2_ molecules through hydrogen bonding and electrostatic interactions.^31,32^ The incorporation of melamine within porous frameworks may therefore enhance low-pressure CO_2_ affinity even at the expense of accessible porosity. Such behavior is particularly relevant for highly diluted CO_2_ streams, where adsorption affinity becomes more critical than maximum adsorption capacity at high pressure.^5,13^

In this work, MIL-101­(Cr) was modified through melamine incorporation to investigate the effect of nitrogen-rich adsorption sites on low-pressure CO_2_ capture behavior. Despite the partial reduction in accessible porosity after melamine incorporation, the modified material exhibited enhanced low-pressure CO_2_ uptake, improved CO_2_/N_2_ selectivity, and superior working capacity under elevated-temperature conditions. Furthermore, isosteric enthalpy calculations and DRIFTS analyses suggest that improved adsorption performance originates from moderate-strength non-covalent CO_2_-melamine interactions rather than from irreversible chemisorptive binding. These results demonstrate that adsorption affinity can play a more critical role than total porosity in determining CO_2_ capture performance under low-pressure conditions.

## Materials and Methods

### Reagents

Cr­(NO_3_)_3_·9H_2_O, terephthalic acid, HF, and melamine were purchased from Sigma-Aldrich and used as received without further purification.

### Porous Material Preparation

#### Synthesis of MIL-101­(Cr)

MIL-101­(Cr) was synthesized through a hydrothermal procedure adapted from a previously reported method.^33^ Briefly, 1.67 g of terephthalic acid (C_6_H_4_(COOH)_2_) and 4.00 g of chromium­(III) nitrate nonahydrate (Cr­(NO_3_)_3_·9H_2_O) were dissolved in 42 mL of deionized water, followed by the addition of 0.5 mL of hydrofluoric acid (HF). The resulting mixture was transferred into a Teflon-lined stainless-steel autoclave and heated at 220 °C for 8 h. After cooling to room temperature, the obtained green solid was recovered, repeatedly washed with ethanol (95%), and dried at 80 °C for 24 h.

#### Melamine Impregnation on MIL-101­(Cr)

Melamine (Figure 1) was incorporated into the activated MIL-101­(Cr) through a wet impregnation procedure. Briefly, 0.050 g of melamine was dissolved in 100 mL of anhydrous ethanol, followed by the addition of 0.200 g of MIL-101­(Cr), corresponding to a nominal melamine loading of 25 wt % relative to the MOF mass. The suspension was stirred at 500 rpm for 4 h at room temperature. Subsequently, the solvent was removed by evaporation at approximately 80 °C. The obtained solid was further dried at 80 °C overnight and stored in a desiccator prior to characterization.

**Figure 1:**
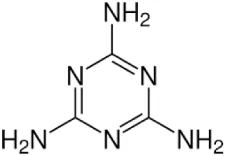
Chemical structure of melamine.

### Porous Material Characterization

#### Characterization Techniques

Powder X-ray diffraction (PXRD) patterns were recorded using a Rigaku MiniFlex II diffractometer (Tokyo, Japan) equipped with Cu Kα radiation (λ = 1.5418 Å) over a 2θ range of 2–40°.

Thermogravimetric analysis (TGA) was carried out using an SDTQ-600 TGA/DSC instrument (TA Instruments) under a nitrogen atmosphere (20 mL min^–1^). Approximately 10 mg of sample was heated from 30 to 1000 °C at a heating rate of 5 °C min^–1^.

Nitrogen adsorption-desorption isotherms were measured at 77 K using a Micromeritics ASAP 2020 system (Norcross, USA). Prior to the measurements, the samples were degassed under vacuum at 120 °C for 18 h. The specific surface area was calculated according to the Brunauer–Emmett–Teller (BET) method following the Rouquerol consistency criteria. Pore size distributions were obtained using the nonlocal density functional theory (NLDFT) method with a nitrogen kernel, while the total pore volume was estimated from the amount adsorbed at a relative pressure (P/P_0_) of 0.99.

### CO_2_ Sorption Measurements

CO_2_ adsorption isotherms were measured in the low-pressure region (0–1 bar) at 0, 30, 45, and 60 °C using a Micromeritics ASAP 2020 system (Norcross, USA). Prior to the measurements, the samples were degassed under vacuum at 120 °C for 18 h to remove pre-adsorbed species from the porous structure.

The isosteric enthalpy of adsorption (−ΔH_ads_) was determined from the CO_2_ adsorption isotherms collected at different temperatures using the Clausius–Clapeyron eq (eq 1).

1 $$\ln P=-\frac{\Delta H}{R}\frac{1}{T}+c$$

where *p* is the equilibrium pressure, *T* is the absolute temperature, and *R* is the universal gas constant (8.314 J mol^–1^ K^–1^). The isosteric enthalpy of adsorption (−ΔH_ads_) was obtained from the slope of the linear plot of ln­(*p*) versus 1/*T*, providing information about the strength of the interactions between CO_2_ molecules and the adsorption sites within the porous structure.

The CO_2_/N_2_ selectivity was predicted using the Ideal Adsorbed Solution Theory (IAST) from the experimentally obtained single-component adsorption isotherms (eq 2).^34^

2 $$S_{ads}=\frac{qCO_{2}/qN_{2}}{pCO_{2}/pN_{2}}$$

where *q*CO_2_ and qN_2_ represent the adsorption capacities of CO_2_ and N_2_ at their respective partial pressures in the binary gas mixture, with *p*CO_2_ = 0.15 bar and *p*N_2_ = 0.85 bar. Single-component adsorption isotherms for CO_2_ and N_2_ were collected at 30 °C in the low-pressure region (0–1 bar).

Pressure swing adsorption (PSA) cyclic stability was evaluated using a Micromeritics 3Flex analyzer (Micromeritics Instrument Corp., USA). Five consecutive CO_2_ adsorption-desorption cycles were performed by alternating the pressure between 0.015 and 1.0 bar.

### Kinetic CO_2_ Adsorption Measurements

Kinetic CO_2_ adsorption experiments were carried out using a Shimadzu DTG-60/60-H thermogravimetric analyzer (Japan) following the procedure reported by Hisse et al. (2024).^35^ Approximately 5 mg of sample was placed in an alumina pan and pretreated at 120 °C under a N_2_ flow of 50 mL min^–1^ for 1 h, or until a constant mass was reached. Subsequently, the temperature was reduced to 30 °C, and a CO_2_ flow of 50 mL min^–1^ was introduced while maintaining a N_2_ flow of 50 mL min^–1^ through the balance side. The CO_2_ uptake was continuously monitored until adsorption equilibrium was achieved. Desorption experiments were initiated by interrupting the CO_2_ flow, and the mass variation was recorded until the sample recovered its initial mass.

### Breakthrough Experiments

Dynamic breakthrough experiments were performed using a Micromeritics 3Flex system coupled to a U-shaped quartz reactor. Approximately 300 mg of MIL-101­(Cr)-MEL was activated under vacuum at 120 °C for 18 h using a Smart VacPrep system. The activated sample was then transferred to the quartz reactor and purged with Ar prior to the breakthrough experiments. The experiments were conducted at 30 °C. He (10 sccm) was used as an inert tracer gas, after which the feed was switched to a binary mixture of CO_2_ (10 sccm) and N_2_ (10 sccm). The outlet gas composition was continuously monitored using an MKS Cirrus 2 mass spectrometer to obtain breakthrough curves. For experiments under humid conditions, the CO_2_/N_2_ gas mixture was passed through a water saturator at room temperature before entering the adsorption bed.

### DRIFTS Analysis of CO_2_ Adsorption

Diffuse reflectance infrared Fourier transform spectroscopy (DRIFTS) measurements were performed using a Bruker Vertex 70 spectrophotometer (Billerica, USA) equipped with a Harrick Praying Mantis accessory and an HVC-DRP-4 reaction chamber fitted with ZnSe windows. Prior to the measurements, the samples were pretreated in situ at 115 °C under a helium flow for 15 min and subsequently cooled to 30 °C. A background interferogram was recorded under continuous helium flow before CO_2_ exposure. The samples were then exposed to a CO_2_ stream for 20 min, after which the gas flow was switched back to helium and the first spectrum was immediately collected. Additional spectra were subsequently acquired at 2 min intervals over a total monitoring period of 20 min. All interferograms were collected by averaging 256 scans with a spectral resolution of 16 cm^–1^.

## Results and Discussion

The successful synthesis of MIL-101­(Cr) was confirmed by PXRD, N_2_ adsorption at 77 K, and thermogravimetric analyses. The PXRD pattern (Figure S1a) agrees well with previously reported MIL-101­(Cr) structures, confirming the formation of a highly crystalline framework.^36,37^ The pristine MIL-101­(Cr) exhibited a BET surface area of 3303 m^2^ g^–1^ (Figure S1b), consistent with literature values reported for highly porous MIL-101­(Cr) materials.^21^ Furthermore, the TGA and DTG profiles (Figure S1c) displayed three main weight-loss regions centered around 100, 250, and 400–450 °C, associated with the removal of physisorbed water, desorption of confined species, and framework decomposition into Cr_2_O_3_, respectively.^38^

Figure 2a presents the N_2_ adsorption-desorption isotherms of MIL-101­(Cr) and MIL-101­(Cr)-MEL measured at 77 K. Both materials exhibit type I isotherms according to the IUPAC classification, characteristic of predominantly microporous solids,^39^ while the small hysteresis loop observed at intermediate relative pressures suggests the presence of secondary mesoporosity associated with the MIL-101­(Cr) framework.^40^ The sharp N_2_ uptake at low relative pressures confirms the highly porous nature of both materials.

**Figure 2:**
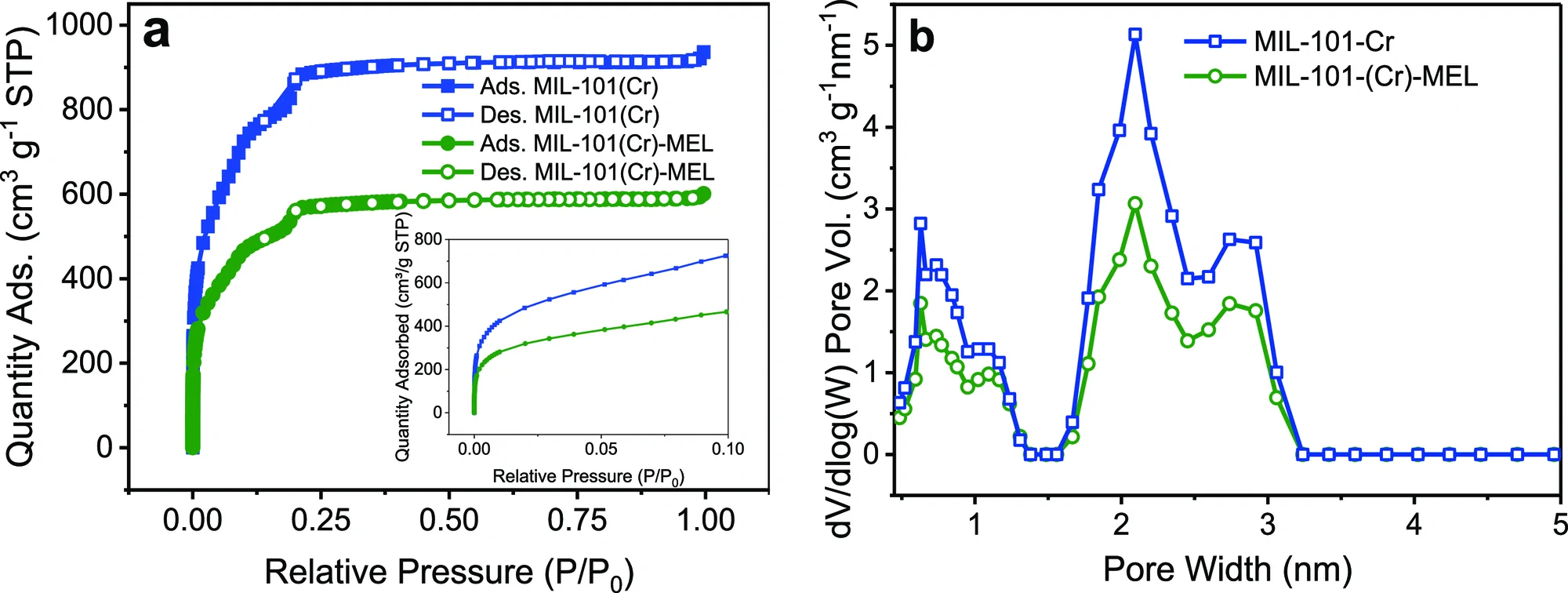
(a) N_2_ adsorption-desorption isotherms at 77 K and (b) pore size distribution profiles of MIL-101­(Cr) and MIL-101­(Cr)-MEL.

The pristine MIL-101­(Cr) exhibited a significantly higher N_2_ uptake and BET surface area than MIL-101­(Cr)-MEL, whose surface area decreased to 2090 m^2^ g^–1^ after melamine incorporation. Similarly, the pore size distribution profiles (Figure 2b) reveal a reduction of approximately 36% in accessible pore volume after modification, while preserving the characteristic pore size distribution of MIL-101­(Cr). These results indicate that melamine incorporation partially occupies the porous structure without causing significant framework collapse or loss of structural integrity. Importantly, although melamine incorporation reduces the accessible porosity of the material, the preservation of the pore structure suggests that adsorption sites remain accessible for gas adsorption under low-pressure conditions.

The marked decrease in accessible pore volume (∼36%) while preserving the characteristic pore size distribution and crystallinity of MIL-101­(Cr) indicates that melamine is primarily confined within the porous network rather than merely deposited on the external surface. Such confinement is expected to be favored by weak host-guest interactions, including possible hydrogen bonding between melamine amino groups and oxygen atoms of the framework.

Water adsorption isotherms (Figure 3) reveal a significant decrease in H_2_O uptake after melamine incorporation into MIL-101­(Cr). While the pristine material exhibits a pronounced increase in water adsorption at moderate-to-high relative pressures, MIL-101­(Cr)-MEL shows substantially lower uptake over the entire pressure range, reaching a maximum adsorption capacity of approximately 52 mmol g^–1^. The high-water uptake of pristine MIL-101­(Cr) is associated with its large accessible pore volume and hydrophilic adsorption sites within the framework. After melamine incorporation, the reduced H_2_O adsorption is mainly attributed to partial pore occupation and decreased accessibility of water molecules to the porous structure. This lower water uptake may be beneficial for low-pressure CO_2_ capture applications, where competitive adsorption between H_2_O and CO_2_ can negatively affect adsorption performance.

**Figure 3:**
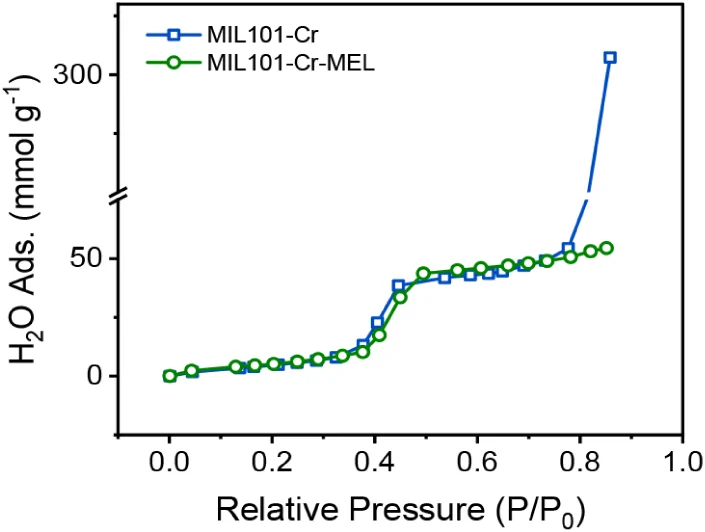
Water adsorption isotherms of MIL-101­(Cr) and MIL-101­(Cr)-MEL.

The PXRD patterns of theoretical MIL-101­(Cr), as-synthesized MIL-101­(Cr), and MIL-101­(Cr)-MEL are presented in Figure 4. The diffraction pattern of the synthesized material agrees well with the theoretical MIL-101­(Cr) structure, confirming the successful formation of the crystalline framework. After melamine incorporation, the main characteristic diffraction peaks of MIL-101­(Cr) remain largely preserved, indicating that the overall framework structure and crystallinity are maintained after modification. Minor changes in peak intensity and slight peak broadening are observed for MIL-101­(Cr)-MEL, which may be associated with partial pore occupation and local structural modifications within the porous framework.

**Figure 4:**
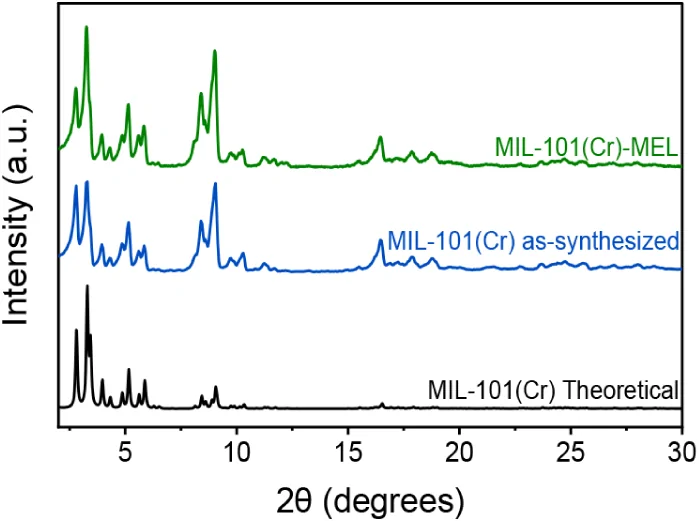
PXRD patterns of theoretical MIL-101­(Cr), as-synthesized MIL-101­(Cr), and MIL-101­(Cr)-MEL.

Figure 5 presents the CO_2_ adsorption isotherms of MIL-101­(Cr) and MIL-101­(Cr)-MEL measured between 0 and 60 °C in the low-pressure region (0–1 bar). At 0 °C, pristine MIL-101­(Cr) exhibits the highest CO_2_ uptake at pressures approaching 1 bar, reaching approximately 4.0 mmol g^–1^, whereas MIL-101­(Cr)-MEL reaches approximately 3.3 mmol g^–1^. This behavior is consistent with the larger accessible surface area and pore volume of pristine MIL-101­(Cr). However, despite its lower total uptake, MIL-101­(Cr)-MEL displays a steeper adsorption profile in the low-pressure region, indicating the presence of higher-affinity adsorption sites after melamine incorporation.

**Figure 5:**
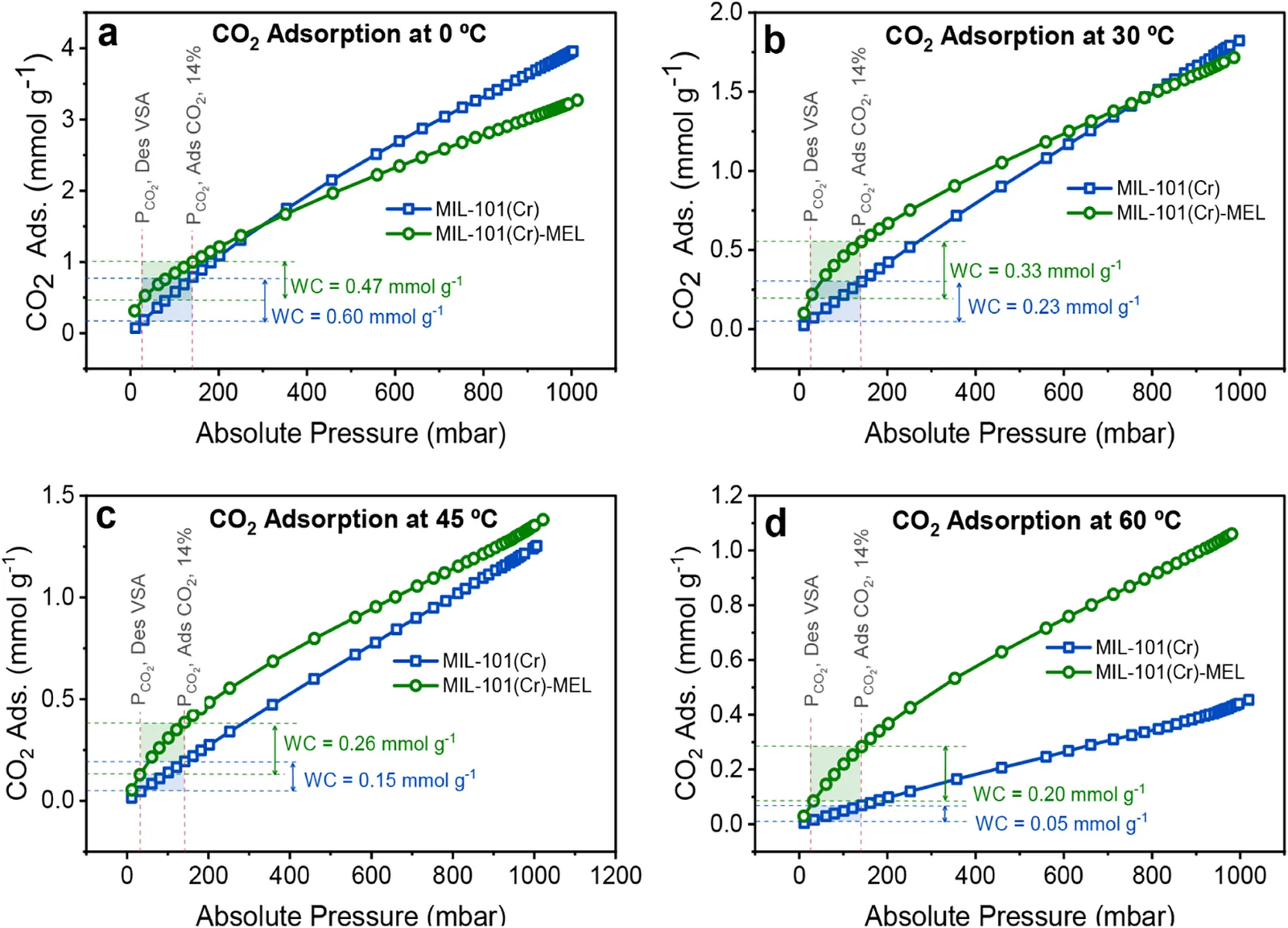
CO_2_ adsorption isotherms of MIL-101­(Cr) and MIL-101­(Cr)-MEL measured at (a) 0 °C, (b) 30 °C, (c) 45 °C, and (d) 60 °C.

As temperature increases, the adsorption capacity of pristine MIL-101­(Cr) decreases sharply, reflecting the strong temperature dependence of physisorption-dominated adsorption. In contrast, MIL-101­(Cr)-MEL retains substantially higher CO_2_ uptake, particularly at low pressures and elevated temperatures. At 60 °C, MIL-101­(Cr)-MEL reaches a CO_2_ uptake of 1.06 mmol g^–1^ at 1 bar, whereas pristine MIL-101­(Cr) adsorbs only 0.45 mmol g^–1^. These results indicate that melamine incorporation modifies the dominant adsorption behavior of MIL-101­(Cr), shifting the adsorption process from porosity-dominated uptake toward affinity-controlled adsorption under diluted CO_2_ conditions.

The working capacities (WC) calculated within the 0.08–0.14 bar pressure range are presented in Figure 6, as this interval is relevant for low-pressure CO_2_ capture applications.^41^ While pristine MIL-101­(Cr) exhibits a slightly higher WC at 0 °C, MIL-101­(Cr)-MEL progressively outperforms the pristine material as temperature increases. At 60 °C, MIL-101­(Cr)-MEL retains a WC of 0.20 mmol g^–1^, compared with only 0.05 mmol g^–1^ for pristine MIL-101­(Cr), corresponding to a fourfold enhancement after melamine incorporation. This improved low-pressure adsorption performance at elevated temperature highlights the importance of introducing high-affinity adsorption sites for efficient CO_2_ capture under thermally demanding conditions.

**Figure 6:**
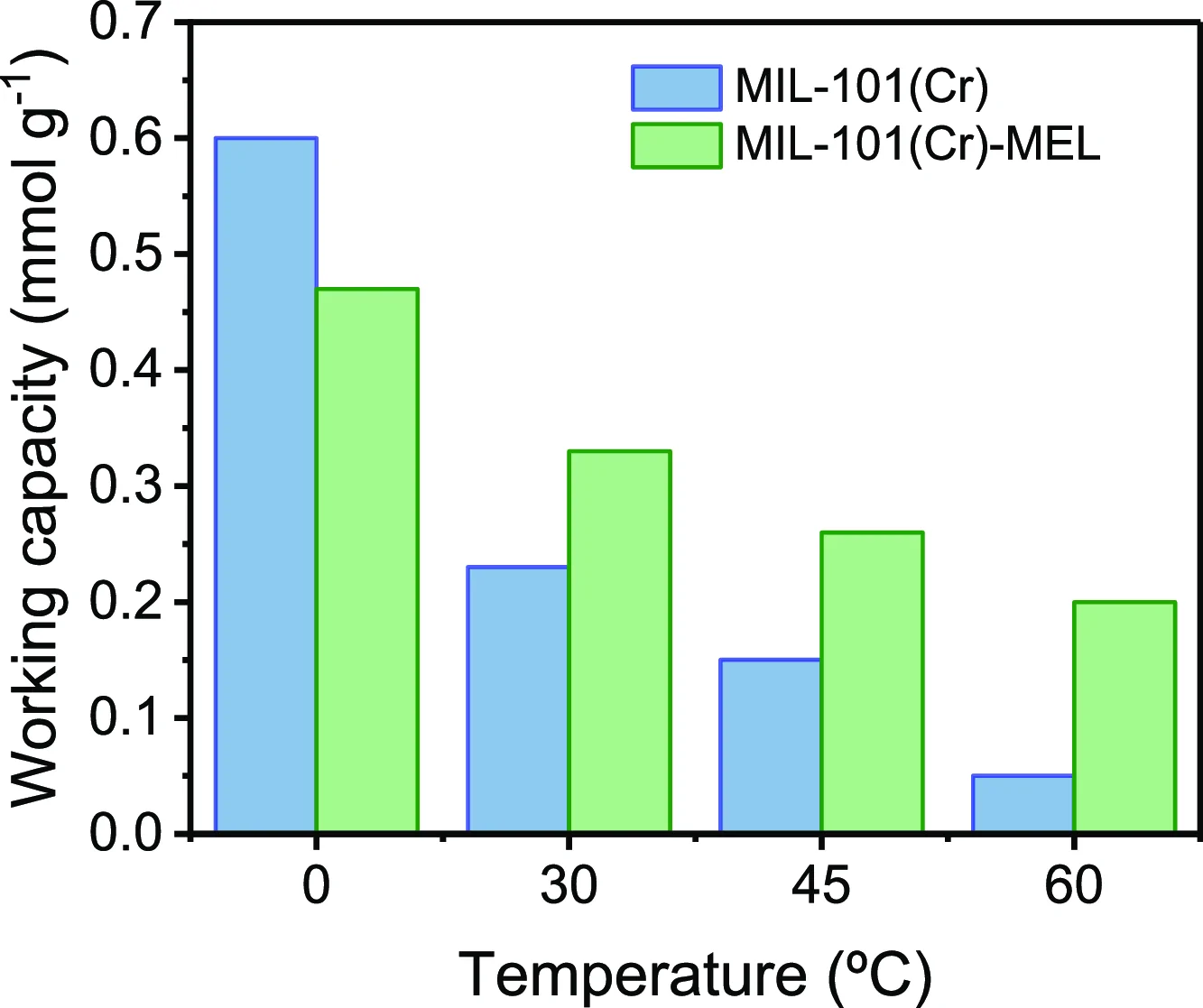
Low-pressure CO_2_ working capacities of MIL-101­(Cr) and MIL-101­(Cr)-MEL within the 0.08–0.14 bar pressure range at 0, 30, 45, and 60 °C.

Importantly, the enhanced low-pressure CO_2_ affinity of MIL-101­(Cr)-MEL suggests that adsorption performance becomes increasingly governed by adsorption affinity rather than total porosity under diluted CO_2_ conditions. The steeper initial uptake and reduced temperature sensitivity observed for the modified material further indicate that melamine incorporation promotes more effective CO_2_ capture under low-pressure conditions.

The CO_2_/N_2_ selectivity was evaluated using IAST^34^ based on single-component adsorption isotherms fitted with Langmuir, Freundlich, and dual-site Langmuir–Freundlich (DSLF) models (Figures S2 and S3). The DSLF model provided the best agreement with experimental data and was therefore used for selectivity calculations.

As shown in Figure 7, MIL-101­(Cr)-MEL exhibits a remarkably high CO_2_/N_2_ selectivity at very low pressures, reaching values close to 90 below 0.05 bar, which is substantially higher than pristine MIL-101­(Cr) (∼30). This behavior indicates that melamine incorporation introduces high-affinity adsorption sites that strongly favor CO_2_ adsorption under diluted CO_2_ conditions. As pressure increases, the selectivity of MIL-101­(Cr)-MEL decreases progressively and stabilizes near ∼19, suggesting progressive saturation of the high-affinity adsorption sites. In contrast, pristine MIL-101­(Cr) exhibits a weaker pressure dependence, consistent with adsorption dominated mainly by weak physisorptive interactions. Overall, these results further support that melamine incorporation shifts the adsorption behavior of MIL-101­(Cr) from porosity-dominated adsorption toward affinity-controlled CO_2_ capture at low pressures.

**Figure 7:**
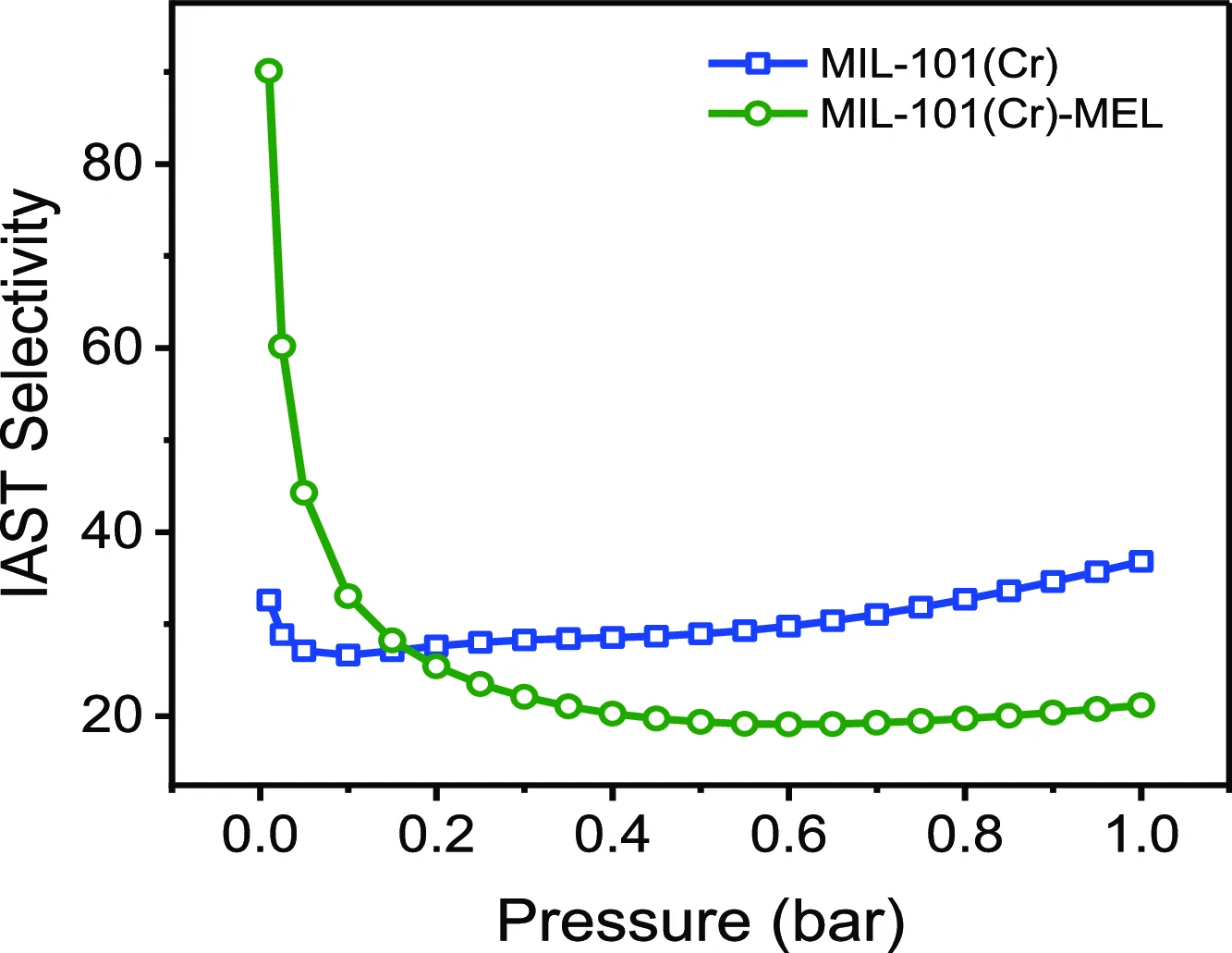
CO_2_/N_2_IAST selectivity of MIL-101­(Cr) and MIL-101­(Cr)-MEL as a function of pressure at 30 °C.

The isosteric enthalpy of CO_2_ adsorption (−ΔH_ads_) was calculated using the Clausius–Clapeyron equation from adsorption isotherms collected at 0, 30, and 45 °C. The corresponding isostere plots used for the calculations are presented in Figures S4 and S5, while the resulting −ΔH_ads_ profiles are shown in Figure 8.

**Figure 8:**
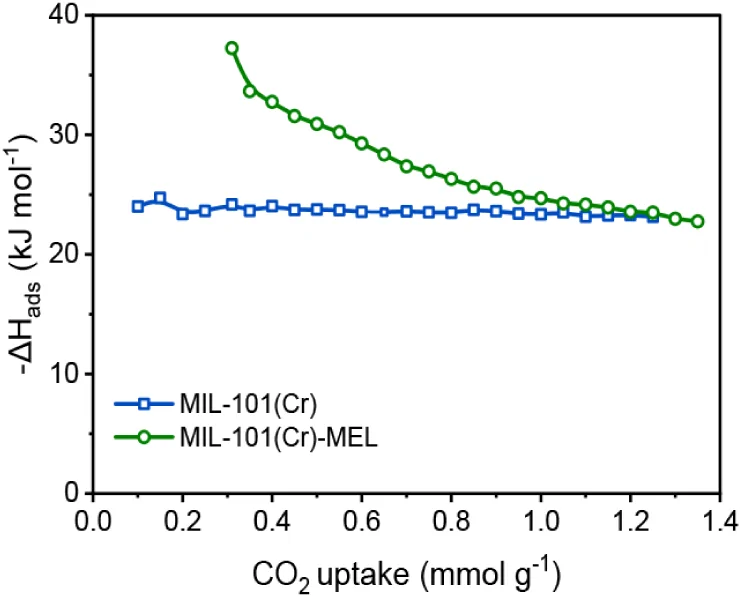
Isosteric enthalpy of CO_2_ adsorption (−ΔH_ads_) of MIL-101­(Cr) and MIL-101­(Cr)-MEL as a function of CO_2_ uptake.

Pristine MIL-101­(Cr) exhibits nearly constant −ΔH_ads_ values of approximately 23–24 kJ mol^–1^ over the entire loading range, which is characteristic of adsorption dominated mainly by weak physisorptive interactions. In contrast, MIL-101­(Cr)-MEL displays significantly higher −ΔH_ads_ values at low CO_2_ loadings, reaching ∼37 kJ mol^–1^, followed by a progressive decrease toward ∼23 kJ mol^–1^ as the CO_2_ uptake increases.

The higher initial enthalpy observed for MIL-101­(Cr)-MEL indicates the presence of stronger adsorption sites introduced after melamine incorporation, which preferentially interact with CO_2_ molecules at low coverage. The progressive decrease in −ΔH_ads_ with increasing CO_2_ uptake suggests gradual saturation of these high-affinity adsorption sites, after which adsorption becomes increasingly dominated by weaker interactions similar to those observed for pristine MIL-101­(Cr). This behavior is consistent with the enhanced low-pressure CO_2_ uptake and high CO_2_/N_2_ selectivity observed for MIL-101­(Cr)-MEL, further supporting that melamine incorporation promotes affinity-controlled CO_2_ adsorption under diluted CO_2_ conditions.

Figure 9a shows the dynamic CO_2_ uptake profiles of MIL-101­(Cr) and MIL-101­(Cr)-MEL obtained under thermogravimetric adsorption conditions. Both materials exhibit rapid CO_2_ uptake upon exposure to the gas stream, followed by a gradual approach to equilibrium. Pristine MIL-101­(Cr) reaches a slightly higher equilibrium adsorption capacity (∼1.0 mmol g^–1^) than MIL-101­(Cr)-MEL (∼0.95 mmol g^–1^), which is consistent with its larger accessible pore volume.

**Figure 9:**
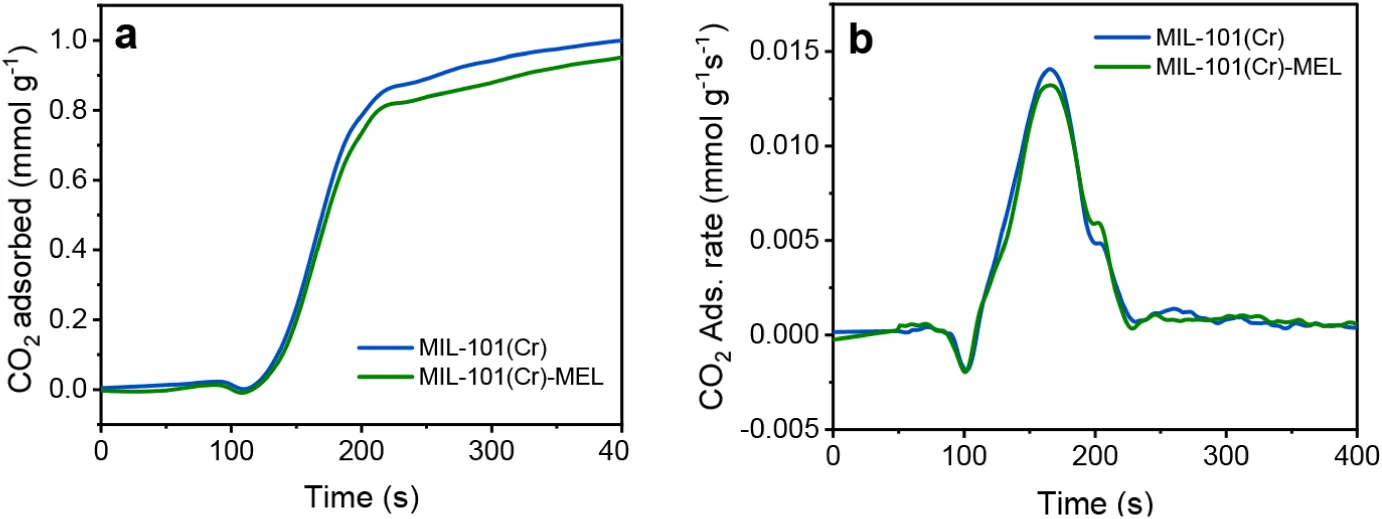
(a) Dynamic CO_2_ uptake profiles and (b) derivative adsorption rate curves of MIL-101­(Cr) and MIL-101­(Cr)-MEL obtained from thermogravimetric adsorption measurements.

The corresponding derivative adsorption rate curves (Figure 9b) reveal comparable adsorption kinetics for both materials, with maximum adsorption rates occurring between approximately 140 and 170 s. A slight reduction in the maximum adsorption rate is observed for MIL-101­(Cr)-MEL; however, both materials exhibit rapid CO_2_ uptake and reach equilibrium within a similar time interval. These results indicate that melamine incorporation enhances low-pressure CO_2_ affinity while preserving the fast adsorption kinetics and accessibility of the porous structure.

Figure 10a shows five consecutive PSA cycles for CO_2_ on MIL-101­(Cr)-MEL. The adsorption capacity remained nearly constant throughout the repeated adsorption-desorption cycles, with no noticeable loss in CO_2_ uptake. The complete regeneration achieved after each desorption step indicates that the adsorbed CO_2_ can be readily released under the applied PSA conditions, demonstrating the excellent cyclic stability and regenerability of the adsorbent.

**Figure 10:**
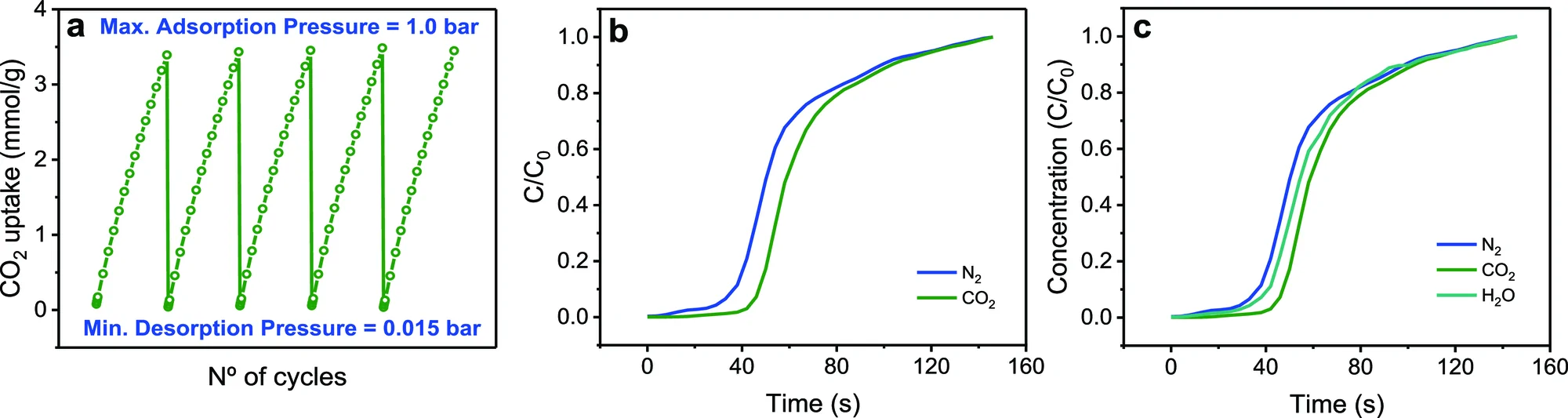
(a) PSA cyclic CO_2_ adsorption-desorption performance of MIL-101­(Cr)-MEL, (b) binary CO_2_/N_2_ breakthrough curves, and (c) CO_2_/N_2_ breakthrough under humid conditions.

To experimentally validate the CO_2_/N_2_ selectivity predicted by IAST (Figure 7), dynamic breakthrough experiments were performed using a binary CO_2_/N_2_ gas mixture. As shown in Figure 10b, N_2_ broke through the adsorption bed before CO_2_, whereas the delayed CO_2_ breakthrough indicates its stronger affinity for the adsorption sites of MIL-101­(Cr)-MEL. This breakthrough behavior is consistent with the equilibrium adsorption results and experimentally confirms the high CO_2_/N_2_ selectivity predicted by IAST.

The dynamic breakthrough curves obtained under humid CO_2_/N_2_ conditions are presented in Figure 10c. Compared with the dry condition, the presence of water vapor results in a slight decrease in CO_2_ adsorption capacity (13.4%), which can be attributed to competitive adsorption between H_2_O and CO_2_ molecules. However, MIL-101­(Cr)-MEL maintains preferential CO_2_ adsorption, as evidenced by the delayed CO_2_ breakthrough relative to N_2_. This behavior is consistent with the lower H_2_O uptake observed after melamine incorporation (Figure 3), which results from partial pore occupation and reduced accessibility of hydrophilic adsorption sites, thereby limiting water adsorption and improving the humidity tolerance of the MIL-101­(Cr) modified with melamine.

The DRIFTS spectra of MIL-101­(Cr) and MIL-101­(Cr)-MEL, including the characteristic bands associated with melamine incorporation, are provided in Figure S6. The spectra of both materials recorded before and after CO_2_ exposure are presented in Figure S7, while the corresponding difference spectra highlighting the physisorption and chemisorption regions are shown in Figure S8.

Figure 11 presents the most relevant spectral regions associated with CO_2_ adsorption, corresponding to the physisorption (2400–2300 cm^–1^) and chemisorption-related/framework-perturbation (1700–1100 cm^–1^) domains. In the 2400–2300 cm^–1^ region (Figure 11a,c,e), both MIL-101­(Cr) and MIL-101­(Cr)-MEL exhibit the characteristic splitting of the *ν*_3_ asymmetric stretching mode of adsorbed CO_2_, which is commonly associated with physisorbed CO_2_ confined within porous frameworks. The preservation of these bands indicates that CO_2_ adsorption remains predominantly reversible and dominated by confinement effects within the porous structure.

**Figure 11:**
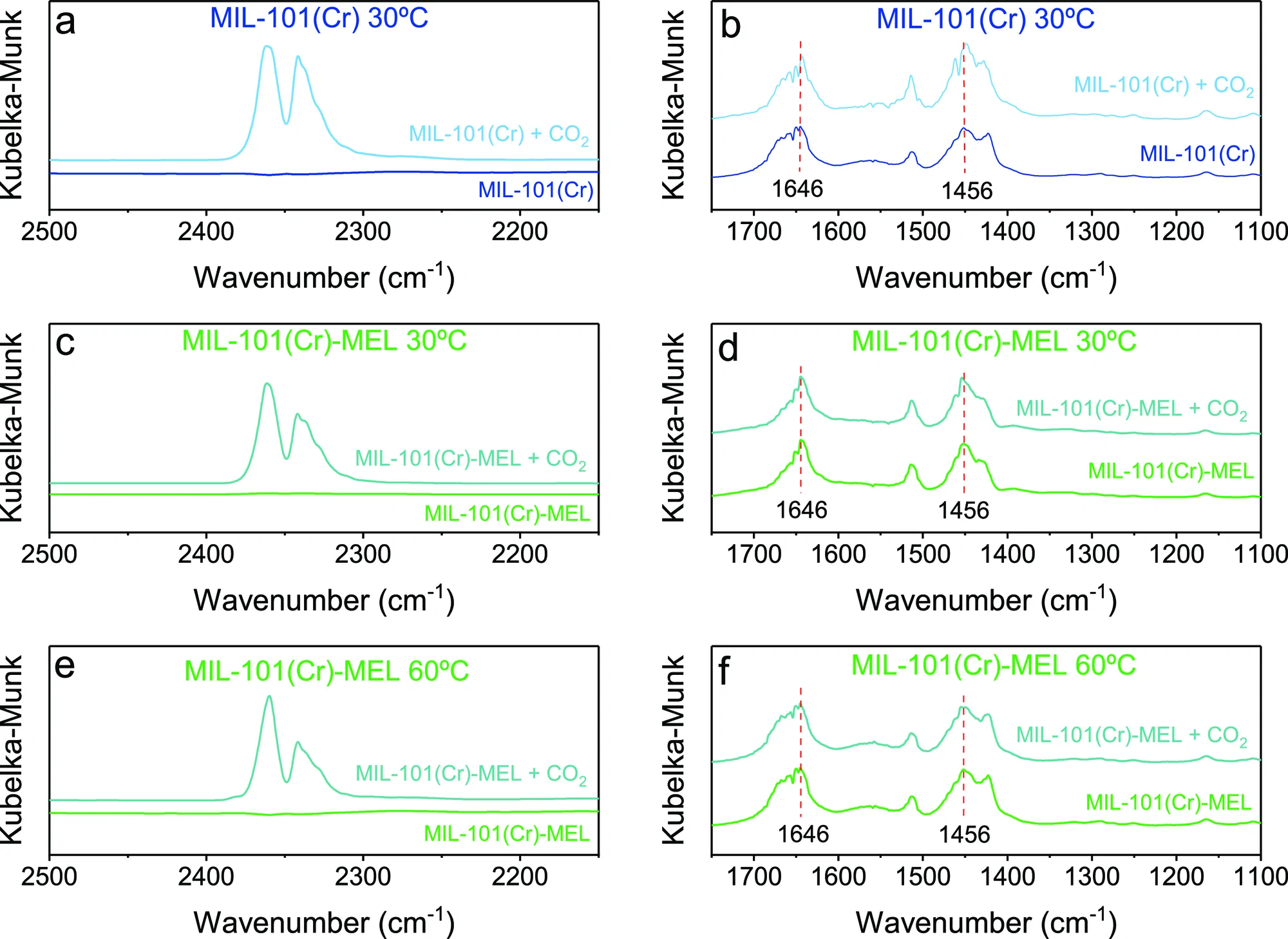
DRIFTS difference spectra of MIL-101­(Cr) and MIL-101­(Cr)-MEL after CO_2_ adsorption at different temperatures: (a) MIL-101­(Cr) at 30 °C in the physisorption region; (b) MIL-101­(Cr) at 30 °C in the chemisorption-related/framework-perturbation region; (c) MIL-101­(Cr)-MEL at 30 °C in the physisorption region; (d) MIL-101­(Cr)-MEL at 30 °C in the chemisorption-related/framework-perturbation region; (e) MIL-101­(Cr)-MEL at 60 °C in the physisorption region; and (f) MIL-101­(Cr)-MEL at 60 °C in the chemisorption region.

More significant differences between both materials are observed in the 1700–1400 cm^–1^ region (Figure 11b, d, f). For pristine MIL-101­(Cr), the bands centered near 1646 and 1456 cm^–1^ exhibit slight shifts toward lower wavenumbers after CO_2_ exposure, indicating weak perturbations of the framework environment caused by physisorbed CO_2_ molecules. In contrast, MIL-101­(Cr)-MEL exhibits distinct spectral behavior, particularly for the band near 1456 cm^–1^, which shifts toward higher wavenumbers after CO_2_ adsorption at both 30 and 60 °C. These differences suggest that melamine incorporation modifies the local electrostatic environment of the porous framework and promotes stronger interactions between CO_2_ molecules and the adsorption sites.

Importantly, no new bands associated with carbonate, bicarbonate, or carbamate species were observed after CO_2_ exposure, even at 60 °C. This indicates that CO_2_ adsorption in MIL-101­(Cr)-MEL is not dominated by irreversible chemisorption, but rather by moderate-strength interactions capable of enhancing low-pressure CO_2_ affinity while preserving reversible adsorption behavior. These observations are fully consistent with the enhanced low-pressure CO_2_ uptake, high CO_2_/N_2_ selectivity, and increased −ΔH_ads_ values observed for MIL-101­(Cr)-MEL.

Although no spectroscopic evidence of carbonate or carbamate formation was observed, the enhanced low-pressure CO_2_ adsorption behavior of MIL-101­(Cr)-MEL suggests the presence of specific interactions between melamine functionalities and CO_2_ molecules.

Previous density functional theory (DFT) studies^32^ have shown that melamine interacts with CO_2_ mainly through non-covalent interactions involving distinct roles for the triazine ring nitrogen atoms and the amino (−NH_2_) groups. The pyridine-like nitrogen atoms of the triazine ring act as Lewis base sites, donating electron density to the electrophilic carbon atom of CO_2_ through a tetrel bond with an N···C distance of approximately 2.83 Å. At the same time, the −NH_2_ groups act as hydrogen-bond donors, forming two nearly symmetrical NH···O hydrogen bonds with the oxygen atoms of CO_2_, each with H···O distances of approximately 2.24 Å (Figure 12).^32^

**Figure 12:**
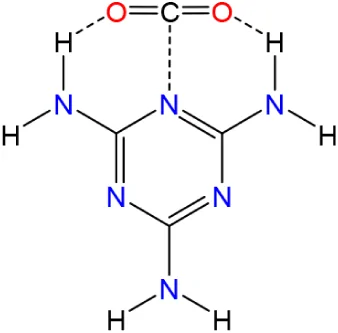
Proposed non-covalent CO_2_-melamine interactions.

These interactions are characterized by moderate stabilization energies and do not involve the formation of covalent CO_2_-amine adducts, which is consistent with the reversible adsorption behavior, moderate −ΔH_ads_ values, and preserved adsorption kinetics observed for MIL-101­(Cr)-MEL.

The adsorption enthalpy comparison presented in Figure 13 and Table S1, including representative amine-modified MIL-100­(Cr) and MIL-101­(Cr) adsorbents from the literature, further supports this interpretation. The moderate −ΔH_ads_ values of MIL-101­(Cr)-MEL suggest a balance between CO_2_ affinity and reversibility, avoiding both the low affinity and strong temperature dependence typical of weakly physisorptive materials and the high regeneration penalties often associated with strong chemisorption. Such a balance represents a highly desirable characteristic for practical low-pressure CO_2_ capture applications.

**Figure 13:**
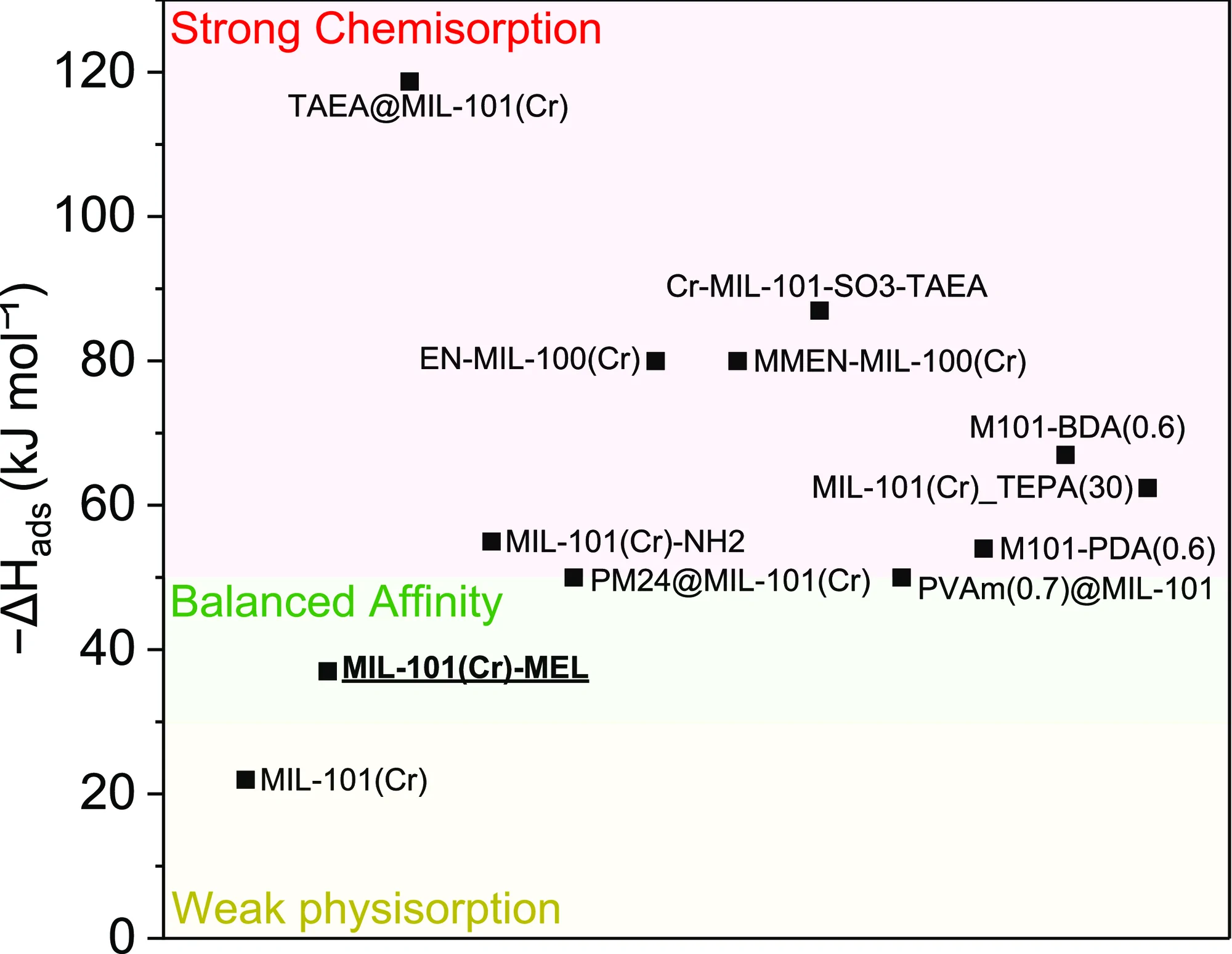
Comparison of isosteric enthalpy of CO_2_ adsorption for amine-functionalized MIL-101­(Cr)/MIL-100­(Cr)-based adsorbents, using values reported in the literature.^42–49^

## Conclusions

Melamine incorporation into MIL-101­(Cr) was successfully achieved while preserving the crystallinity and structural stability of the framework. Although melamine functionalization reduced the accessible porosity of the material, the modified MIL-101­(Cr)-MEL exhibited enhanced CO_2_ adsorption performance under low-pressure and elevated-temperature conditions. In particular, MIL-101­(Cr)-MEL showed significantly improved working capacity and CO_2_/N_2_ selectivity in the low-pressure region, together with reduced temperature sensitivity compared with pristine MIL-101­(Cr).

The increased isosteric enthalpy of adsorption and the DRIFTS analyses indicate that melamine incorporation introduces moderate-strength adsorption sites capable of promoting stronger and more selective interactions with CO_2_ molecules without evidence of irreversible chemisorption. These interactions enhance low-pressure CO_2_ affinity while preserving reversible adsorption behavior and rapid adsorption kinetics.

Overall, the results demonstrate that improving adsorption affinity can be more effective than maximizing total porosity for CO_2_ capture under diluted CO_2_ conditions. Therefore, melamine functionalization represents a simple and effective post-synthetic strategy for tuning the adsorption behavior of MIL-101­(Cr)-based materials toward affinity-controlled CO_2_ capture applications.

## Supplementary Material



## Notes

PXRD pattern, N2 physisorption isotherm, pore size distribution, and TGA/DTG profiles of pristine MIL-101­(Cr) (Figure S1); adsorption isotherm fitting of CO_2_ and N_2_ on MIL-101­(Cr) using Langmuir, Freundlich, and dual-site Langmuir–Freundlich models (Figure S2); adsorption isotherm fitting of CO_2_ and N_2_ on MIL-101­(Cr)-MEL using the same models (Figure S3); isostere plots for CO_2_ adsorption on MIL-101­(Cr) and MIL-101­(Cr)-MEL (Figures S4 and S5); DRIFTS spectra of MIL-101­(Cr) and MIL-101­(Cr)-MEL before and under CO_2_ atmosphere (Figures S6–S8); comparison of amine-functionalized adsorbents and isosteric heats of CO_2_ adsorption at zero coverage (Table S1) (PDF)

## References

[1] Nunes L. J. R. (2023). The
Rising Threat of Atmospheric CO_2_: A Review on the Causes,
Impacts, and Mitigation Strategies. Environments.

[2] Panepinto D., Riggio V. A., Zanetti M. (2021). Analysis of
the Emergent Climate
Change Mitigation Technologies. Int. J. Environ.
Res. Public Health.

[3] Shen M., Kong F., Tong L., Luo Y., Yin S., Liu C., Zhang P., Wang L., Chu P. K., Ding Y. (2022). Carbon Capture
and Storage (CCS): Development Path Based on Carbon Neutrality and
Economic Policy. Carb Neutrality.

[4] Rashid H., Rafey A. (2023). Solid adsorbents for
carbon dioxide capture: a review. Chem. Ecol.

[5] Surkatti R., Abdullatif Y. M., Muhammad R., Sodiq A., Mroue K., Al-Ansari T., Amhamed A. I. (2025). Comparative analysis of amine-functionalized
silica for direct air capture (DAC): Material characterization, performance,
and thermodynamic efficiency. Sep. Purif. Technol.

[6] Bose S., Sengupta D., Malliakas C. D., Idrees K. B., Xie H., Wang X., Barsoum M. L., Barker N. M., Dravid V. P., Islamoglu T., Farha O. K. (2023). Suitability of a diamine functionalized
metal-organic framework for direct air capture. Chem. Sci.

[7] Fan K., Wang Y., Tian C., Guan X., Luo X., Zhou Y. (2025). Metal-organic frameworks for CO_2_ capture: Tailoring structure
and function through modification strategies. Chem. Eng. J.

[8] Usman M., Iqbal N., Noor T., Zaman N., Asghar A., Abdelnaby M. M., Galadima A., Helal A. (2022). Advanced Strategies
in Metal-Organic Frameworks for CO_2_ Capture and Separation. Chem. Rec.

[9] Zulkifli Z. I., Lim K. L., Teh L. P. (2022). Metal-Organic Frameworks (MOFs) and
their Applications in CO_2_ Adsorption and Conversion. ChemistrySelect.

[10] Lancheros A., Goswami S., Zarate X., Blanco E., Schott E., Hupp J. T. (2024). New Pillar-MOF with Nitrogen-Donor Sites for CO_2_ Adsorption. Cryst. Growth Des.

[11] Lancheros A., Goswami S., Mian M. R., Zhang X., Zarate X., Schott E., Farha O. K., Hupp J. T. (2021). Modulation of CO_2_ adsorption in novel pillar-layered MOFs based on carboxylate-pyrazole
flexible linker. Dalton Trans.

[12] Lancheros A., Goswami S., Zarate X., Schott E., Hupp J. T. (2024). Nitrogen-enriched
flexible metal-organic framework for CO_2_ adsorption. Dalton Trans.

[13] Zhang Z., Zheng Y., Qian L., Luo D., Dou H., Wen G., Yu A., Chen Z. (2022). Emerging Trends
in Sustainable CO_2_-Management Materials. Adv. Mater.

[14] Pirzadeh K., Ghoreyshi A. A., Rohani S., Rahimnejad M. (2020). Strong Influence
of Amine Grafting on MIL-101 (Cr) Metal-Organic Framework with Exceptional
CO_2_/N_2_ Selectivity. Ind.
Eng. Chem. Res.

[15] Chong K. C., Ho P. S., Lai S. O., Lee S. S., Lau W. J., Lu S. Y., Ooi B. S. (2022). Solvent-Free Synthesis
of MIL-101­(Cr)
for CO_2_ Gas Adsorption: The Effect of Metal Precursor and
Molar Ratio. Sustainability.

[16] Lee G., Jhung S. H. (2024). Carbon dioxide capture
using diaminoalkane-grafted
MIL-101­(Cr)­s: Critical role of geometric configuration of loaded amines. Sep. Purif. Technol.

[17] Férey C., Mellot-Draznieks C., Serre C., Millange F., Dutour J., Surblé S., Margiolaki I. (2005). Chemistry:
A Chromium Terephthalate-Based
Solid with Unusually Large Pore Volumes and Surface Area. Science.

[18] Fakhraie S., Rajabi H. R., Ghasemy E., Rashidi A., Orooji Y., Hadizadeh M. H., Maklavany D. (2025). Exceptional CO_2_ and H_2_S adsorption
by tuning micro/mesopore ratios with embedded
graphene oxide/N-doped carbon quantum dots in MIL-101­(Cr): experimental
and computational insights. J. Colloid Interface
Sci.

[19] Fakhraie S., Rajabi H. R., Rashidi A., Orooji Y., Ghasemy E., Shayesteh Zeraati A., Rahighi R., Mirhashemi A. (2021). In situ simultaneous
chemical activation and exfoliation of carbon quantum dots for atmospheric
adsorption of H_2_S and CO_2_ at room temperature. Appl. Surf. Sci.

[20] Fakhraie S., Rajabi H. R., Rashidi A. (2023). Fabrication and application of novel
core–shell MIL-101­(Cr)@UiO-66­(Zr) nanocrystals for highly selective
separation of H_2_S and CO_2_. Chem. Eng. J.

[21] Liu Q., Ning L., Zheng S., Tao M., Shi Y., He Y. (2013). Adsorption
of Carbon Dioxide by MIL-101­(Cr): Regeneration Conditions
and Influence of Flue Gas Contaminants. Sci.
Rep.

[22] Jo D., Lee S. K., Cho K. H., Yoon J. W., Lee U. H. (2022). An Amine-Functionalized
Ultramicroporous Metal–Organic Framework for Postcombustion
CO_2_ Capture. ACS Appl. Mater. Interfaces.

[23] Yu J., Chuang S. S. C. (2017). The
Role of Water in CO_2_ Capture by Amine. Ind. Eng. Chem. Res.

[24] Nguyen T. S., Dogan N. A., Lim H., Yavuz C. T. (2023). Amine Chemistry
of Porous CO_2_ Adsorbents. Acc. Chem.
Res.

[25] Said R. B., Kolle J. M., Essalah K., Tangour B., Sayari A. (2020). A Unified
Approach to CO_2_-Amine Reaction Mechanisms. ACS Omega.

[26] Gaikwad S., Kim S. J., Han S. (2019). CO_2_ Capture Using Amine-Functionalized
Bimetallic MIL-101 MOFs and Their Stability on Exposure to Humid Air
and Acid Gases. Microporous Mesoporous Mater.

[27] Wang S., Xie M., Wang S., Zhuo Y. (2025). Kinetic Model describing the effect
of amine loading and temperature on CO_2_ capture by solid
amine adsorbent. Carbon Capture Sci. Technol.

[28] Bose S., Sengupta D., Rayder T. M., Wang X., Kirlikovali K. O., Sekizkardes A. K., Islamoglu T., Farha O. K. (2024). Challenges and Opportunities:
Metal–Organic Frameworks for Direct Air Capture. Adv. Funct. Mater.

[29] Zhao S., Zhang Y., Li L., Feng J., Qiu W., Ning Y., Huang Z., Lin H. (2025). Degradation of amine-functionalized
adsorbents in carbon capture and direct air capture applications:
mechanism and solutions. Sep. Purif. Technol.

[30] Raganati F., Miccio F., Ammendola P. (2021). Adsorption
of Carbon Dioxide for
Post-Combustion Capture: A Review. Energy Fuels.

[31] Li C., Vernuccio S., Moghadam P. Z. (2025). Computational Screening of Amino-Functionalized
Molecules for Direct Air Capture of CO_2_. J. Phys. Chem. A.

[32] Ferrer M., Alkorta I., Elguero J., Oliva-Enrich J. M. (2024). Capture
of CO_2_ by Melamine Derivatives: A DFT Study Combining the
Relative Energy Gradient Method with an Interaction Energy Partitioning
Scheme. J. Phys. Chem. A.

[33] Baeza P., Astudillo C., Diaz M., Matus C., Ramírez M. F., Aguila G., Ojeda J. (2020). Effect of the Incorporation of Ni
in the Adsorption Capacity of Paracetamol (N-Acetyl-P-Aminophenol)
on MIL-101­(Cr). Water, Air, Soil Pollut.

[34] Myers A. L., Prausnitz J. M. (1965). Thermodynamics
of mixed-gas adsorption. AIChE J.

[35] Hisse D., Bessa I. A. A., Silva L. P. C., da Silva A. F. M., Araujo J. R., Archanjo B. S., Soares A. V. H., Passos F. B., Carneiro J. W. M., dos
Santos T. C., Ronconi C. M. (2024). Microporous Nitrogen-Doped
Activated Biochars Derived from Corn: Use of Husk Waste and Urea for
CO_2_ Capture. J. Braz. Chem. Soc.

[36] Zhao T., Li S. H., Shen L., Wang Y., Yang X. Y. (2018). The sized
controlled synthesis of MIL-101­(Cr) with enhanced CO_2_ adsorption
property. Inorg. Chem. Commun.

[37] Li X., Mao Y., Leng K., Ye G., Sun Y., Xu W. (2017). Synthesis
of amino-functionalized MIL-101­(Cr) with large surface area. Mater. Lett.

[38] Adamu U. A., Abu Bakar N. H. H., Zango Z. U., Sambudi N. S., Iqbal A., Hussin M. H., Hamidon T. S. (2023). Low-temperature
synthesis and characterization
of porous chromium terephthalate MIL-101­(Cr) and its photocatalytic
degradation of phenanthrene. Korean J. Chem.
Eng.

[39] Thommes M., Kaneko K., Neimark A. V., Olivier J. P., Rodriguez-Reinoso F., Rouquerol J., Sing K. S. W. (2015). Physisorption of gases, with special
reference to the evaluation of surface area and pore size distribution
(IUPAC Technical Report). Pure Appl. Chem.

[40] Keshta B. E., Yu H., Wang L., Yi H., Jian S., Uddin M. D. A., Ouyang C., Wang Y., Yuan X., Zhang Y., Jin Y., Basit A., Owais Malik M., Awan K. M. (2025). Preparation of unsaturated
MIL-101­(Cr) with Lewis acid sites for the extraordinary adsorption
of anionic dyes. Npj Clean Water.

[41] Larkin C., Li K., Oliva F., García–García F. R. (2024). Scaling
up a hollow fibre adsorption unit for on-board CCS applications using
a real gasoline engine exhaust. Chem. Eng. J.

[42] Rim G., Priyadarshini P., Song M. G., Wang Y., Bai A., Realff M. J., Lively R. P., Jones C. W. (2023). Support Pore Structure
and Composition Strongly Influence the Direct Air Capture of CO_2_ on Supported Amines. J. Am. Chem. Soc.

[43] Lee G., Jhung S. H. (2024). Carbon
dioxide capture using diaminoalkane-grafted
MIL-101­(Cr)­s: critical role of geometric configuration of loaded amines. Sep. Purif. Technol.

[44] Shin S., Yoo D. K., Bae Y. S., Jhung S. H. (2020). Polyvinylamine-loaded
metal-organic framework MIL-101 for effective and selective CO_2_ adsorption under atmospheric or lower pressure. Chem. Eng. J.

[45] Yoo D. K., Yoon T. U., Bae Y. S., Jhung S. H. (2020). Metal-organic framework
MIL-101 loaded with polymethacrylamide with or without further reduction:
Effective and selective CO_2_ adsorption with amino or amide
functionality. Chem. Eng. J.

[46] Li H., Wang K., Feng D., Chen Y. P., Verdegaal W., Zhou H. C. (2016). Incorporation of Alkylamine into Metal-Organic Frameworks
through a Brønsted Acid–Base Reaction for CO_2_ Capture. ChemSuschem.

[47] Cabello C. P., Berlier G., Magnacca G., Rumori P., Palomino G. T. (2015). Enhanced
CO_2_ adsorption capacity of amine-functionalized MIL-100­(Cr)
metal-organic frameworks. CrystEngcomm.

[48] Park J. M., Cha G. Y., Jo D., Cho K. H., Yoon J. W., Hwang Y. K., Lee S. K., Lee U. H. (2022). Amine and fluorine
co-functionalized MIL-101­(Cr) synthesized via a mixed-ligand strategy
for CO_2_ capture under humid conditions. Chem. Eng. J.

[49] Zhong R., Yu X., Meng W., Liu J., Zhi C., Zou R. (2018). Amine-Grafted
MIL-101­(Cr) via Double-Solvent Incorporation for Synergistic Enhancement
of CO_2_ Uptake and Selectivity. ACS
Sustainable Chem. Eng.

