# Supplementary material for: Melamine-Modified MIL-101(Cr) with Enhanced CO2 Affinity for Low-Pressure Capture
Source: ACS Omega. 2026 Sep 15;11(38):57925–34. doi: 10.1021/acsomega.6c07336 (PMC13629403; doi:10.1021/acsomega.6c07336)
Supplement: Supplementary file 1 [file ao6c07336_si_001.pdf]

Supporting Information

Melamine-Modified MIL-101(Cr) with Enhanced CO_2 Affinity for Low-Pressure Capture

Andrés Lancheros[1], Claudio Contreras-Díaz[1], Esteban Camú[1], Thiago C. dos Santos[2], Célia M. Ronconi[3], Néstor Escalona[1], Élodie Blanco[1,4]

[1] *Department of Chemical Engineering and Bioprocesses. Pontificia Universidad Católica de Chile, Santiago, Chile, aflancheros@uc.cl*

[2] *Chemistry Institute, Universidade Federal do Rio de Janeiro, Rio de Janeiro-RJ, Brazil*

[3] *Department of Inorganic Chemistry, Universidade Federal Fluminense, Niterói-RJ, Brazil*

[4] *Department of Engineering and Construction Management. Pontificia Universidad Católica de Chile, Santiago, Chile*

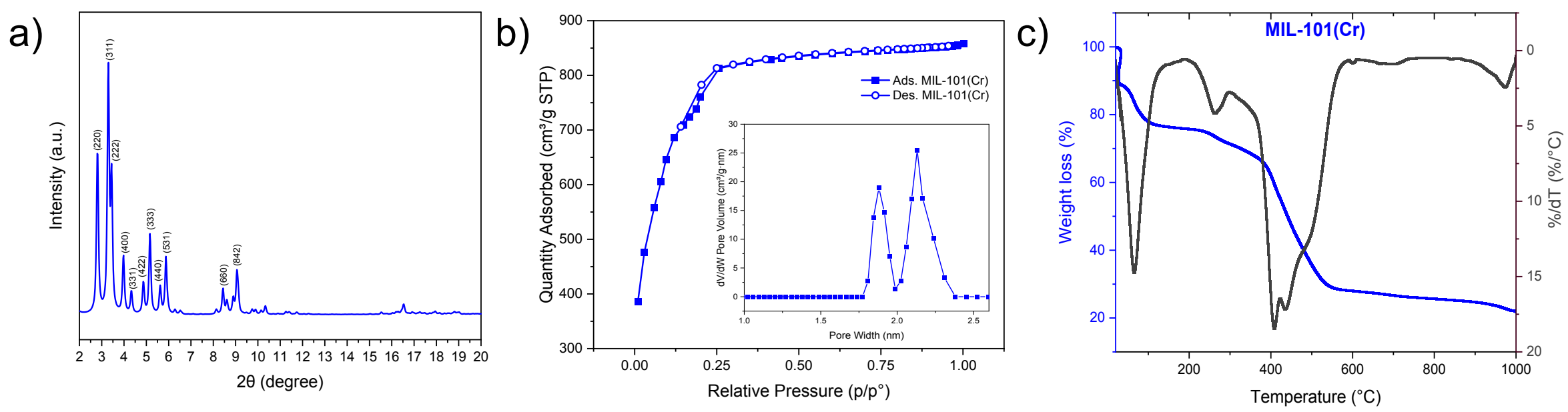

Figure S1. For MIL-101(Cr) a) XRD pattern* b) N_2 physisorption isotherms and pore size distribution and c) thermogravimetric (TGA) and differential (DTG) profiles

*The diffraction peaks of MIL-101(Cr) were observed at 2θ = 2.8° (220), 3.3° (311), 3.4° (222) 4.0° (400), 4.3° (331), 4.9° (422), 5.2° (333), 5.6° (440), 5.9° (531), 6.2° (620), 6.5° (533), 8.1° (733), 8.4° (660), 8.9° (840), 9.1° (842).

The **Langmuir adsorption model** is the simplest theoretical model for monolayer adsorption, which is written as:

$$q_e = \frac{q_m K_L P}{1 + K_L P}$$

where, q_e (mmol/g) represents the amount of the adsorbed gas per unit mass of adsorbent at equilibrium, q_m (mmol/g) is the maximum adsorption capacity, K_L (1/bar) is the Langmuir constant related to the free energy of adsorption and P (bar) is the equilibrium pressure.

Freundlich

$$q_e = K_F P^{\frac{1}{n}}$$

where, q_e (mmol/g) represents the amount of the adsorbed gas per unit mass of adsorbent at equilibrium, kF is Freundlich constant (mmol/g $^{1/n}$) 1/n is the Freundlich exponent, that reflects the degree of heterogeneity of the adsorbent's surface

Dual-site Langmuir Freundlich

$$y = \frac{a_1 b_1 x^{c_1}}{1 + b_1 x^{c_1}} + \frac{a_2 b_2 x^{c_2}}{1 + b_2 x^{c_2}}$$

Where, *y* is the amount adsorbed (mmol/g), *x* is the pressure (bar), *a* is the saturation capacity in mmol/g, b_i is the Langmuir parameter (bar^{-1}) and c_i is the Freundlich parameter for dual sites 1 and 2.

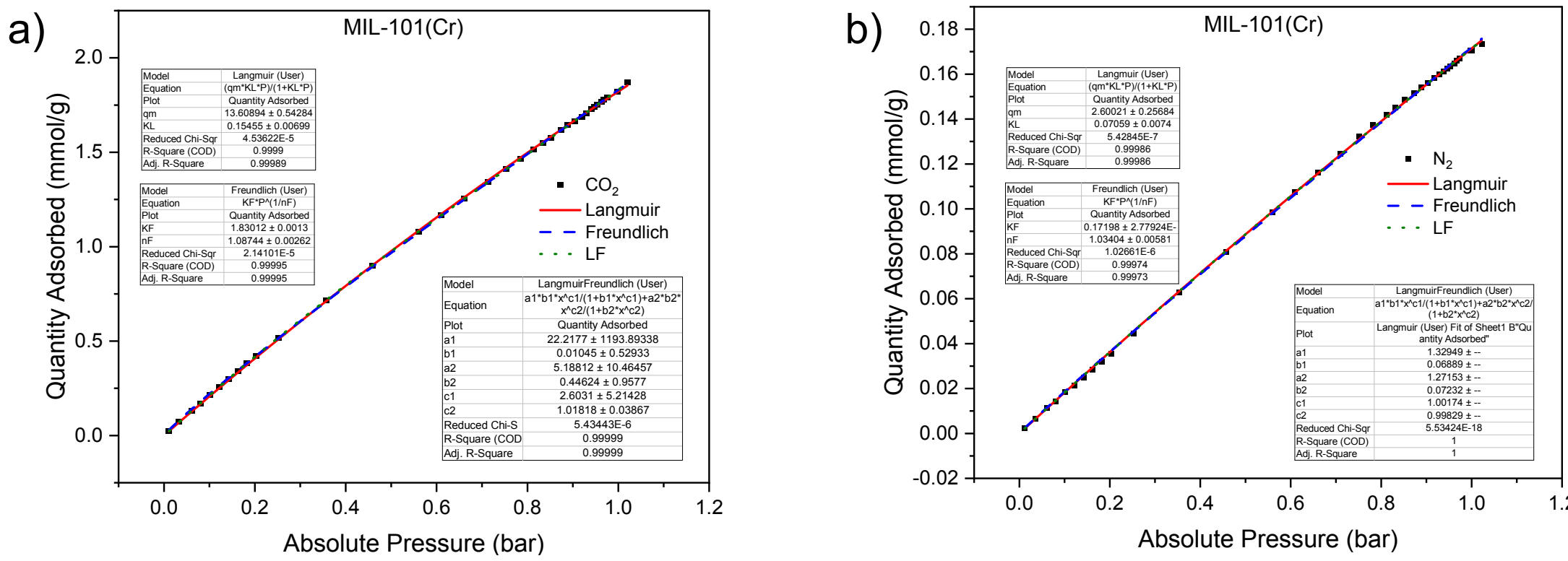

Figure S2. Fitting of MIL-101(Cr) adsorption isotherms using Langmuir, Freundlich, and Dual-Site Langmuir–Freundlich models for (a) CO_2 and (b) N_2

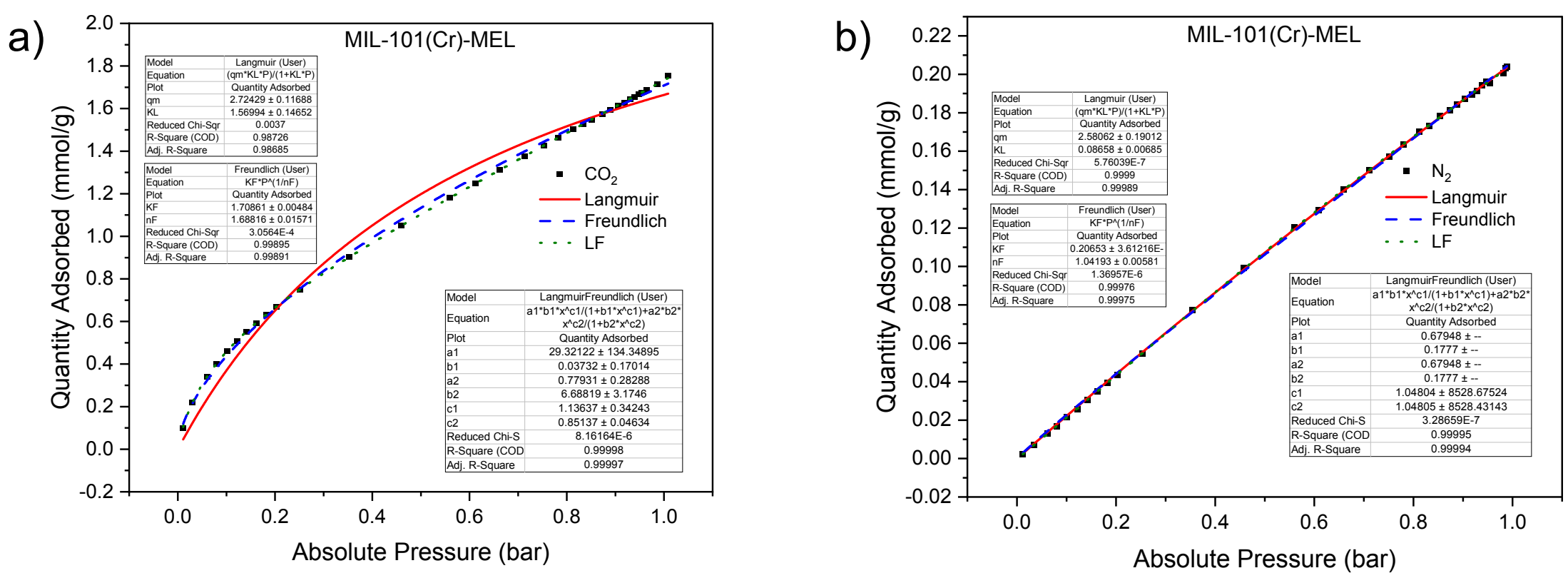

Figure S3. Fitting of MIL-101(Cr)-MEL adsorption isotherms using Langmuir, Freundlich, and Dual-Site Langmuir–Freundlich models for (a) CO_2 and (b) N_2

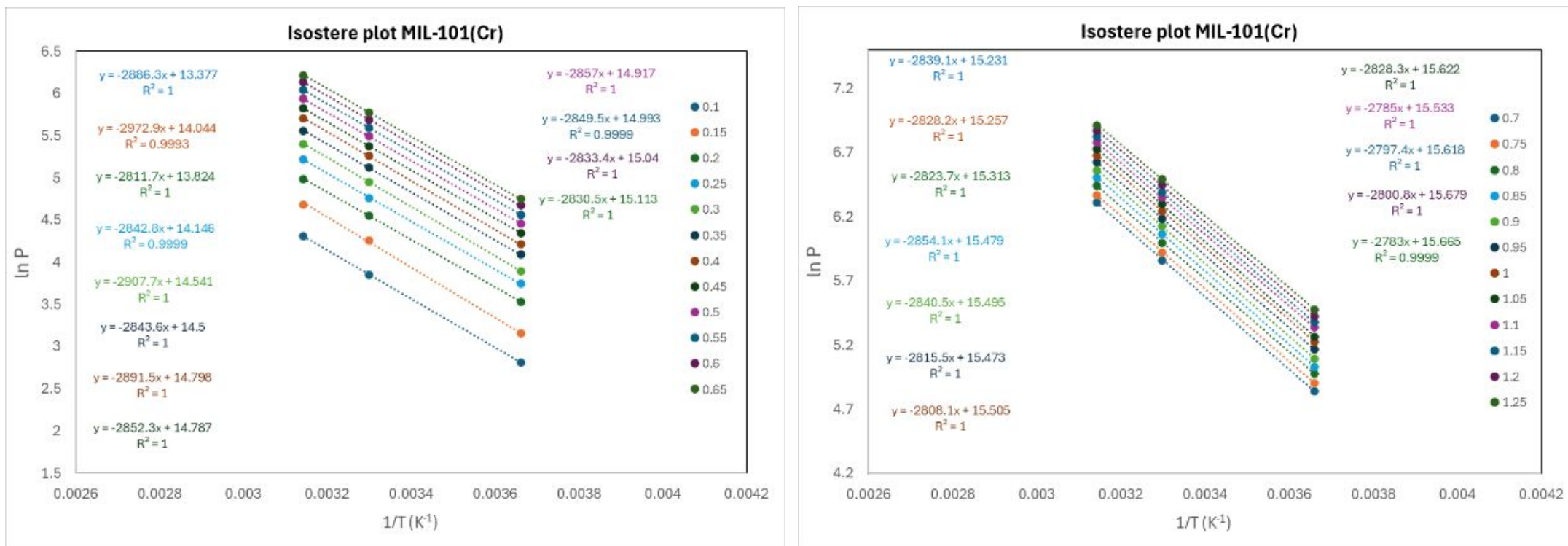

Figure S4. Isostere Plot of CO_2 Adsorption on MIL-101(Cr)

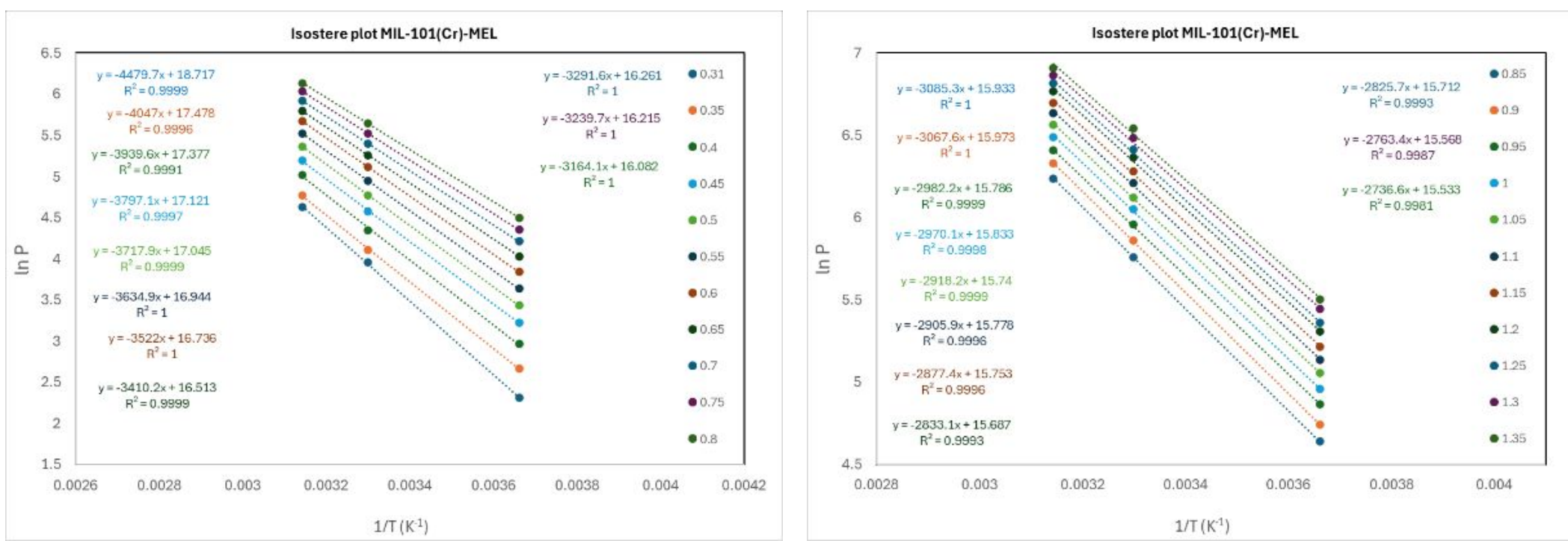

Figure S5. Isostere Plot of CO_2 Adsorption on MIL-101(Cr)-MEL

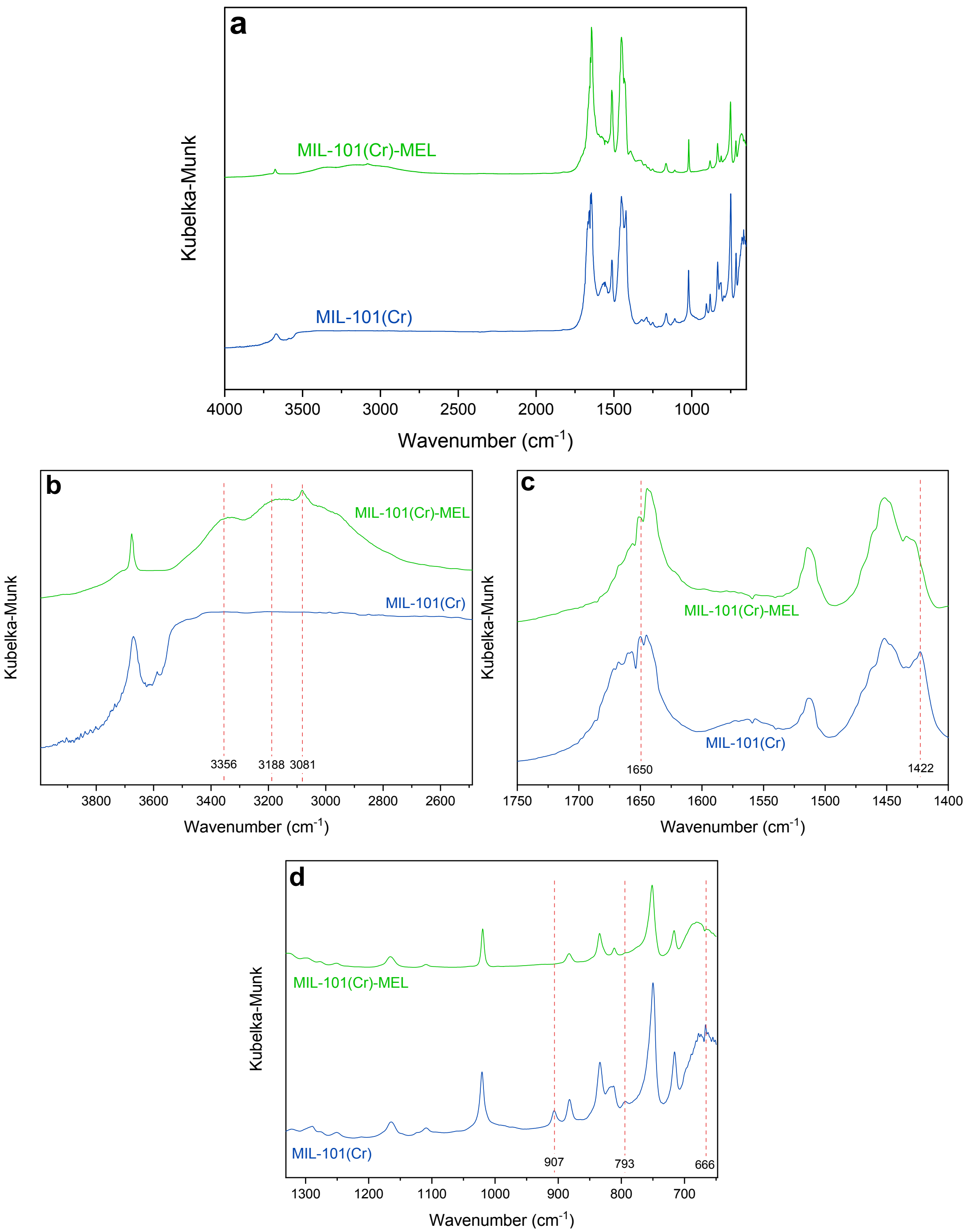

Figure S6. DRIFTS spectra of MIL-101(Cr) and MIL-101(Cr)-MEL: (a) full spectra, (b) zoomed region 3850-2550 cm^{-1}, (c) zoomed region 1750-1400 cm^{-1}, and (d) zoomed region 1350-650 cm^{-1}

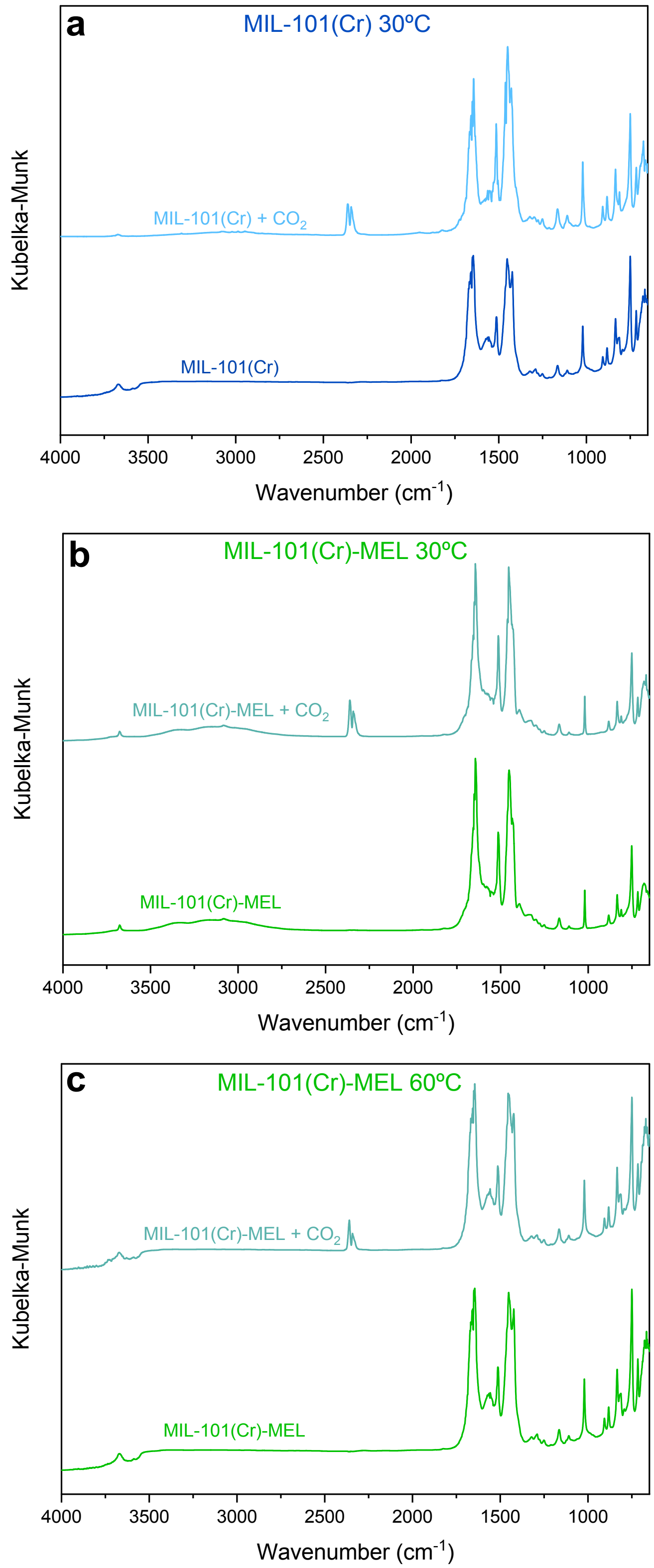

Figure S7. DRIFTS spectra of MIL-101(Cr) and MIL-101(Cr)-MEL under CO_2 atmosphere: (a) MIL-101(Cr) at 30 °C, (b) MIL-101(Cr)-MEL at 30 °C, and (c) MIL-101(Cr)-MEL at 60 °C.

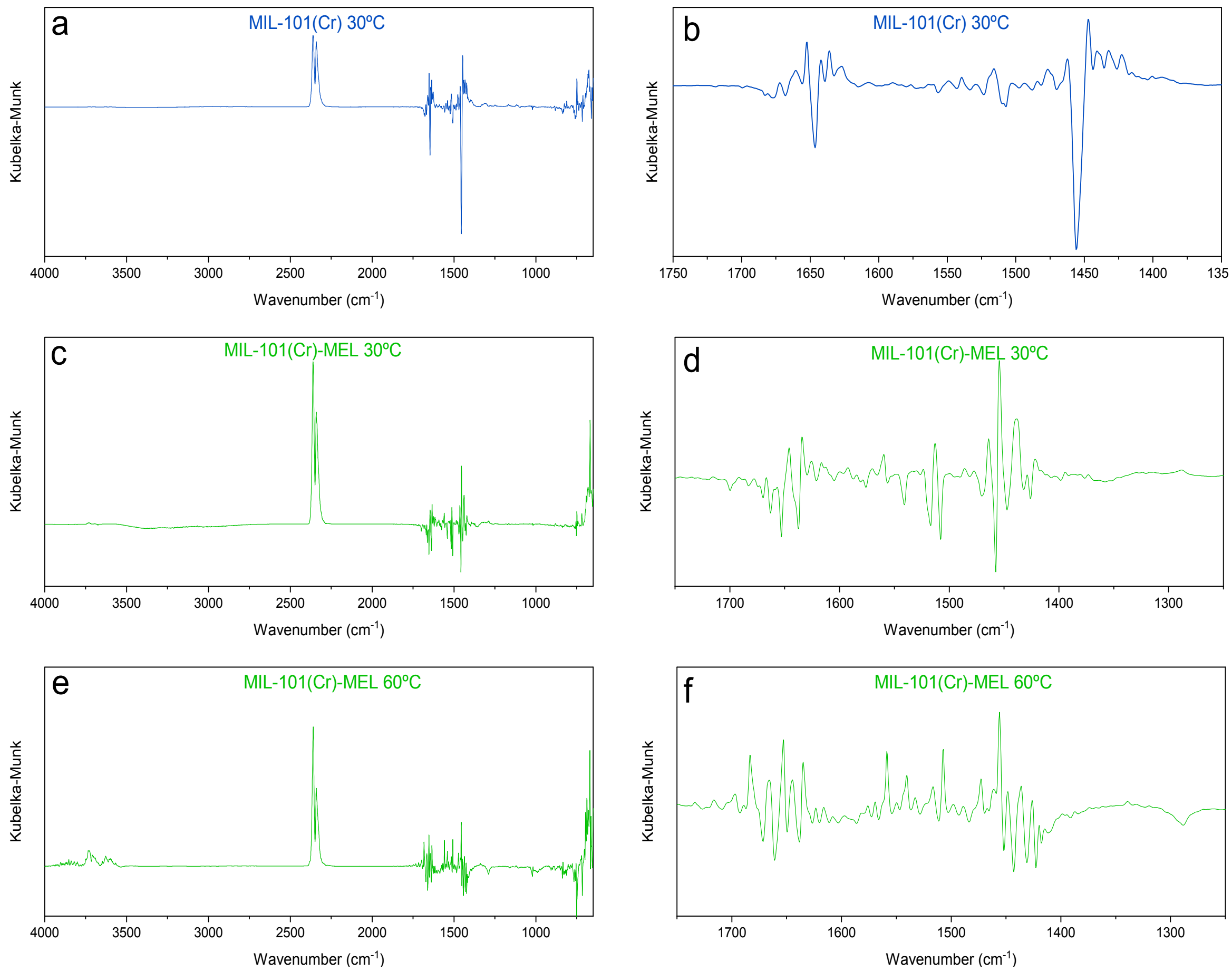

Figure S8. DRIFTS difference spectra obtained by subtracting the background spectrum of the pristine material from that recorded under CO_2 atmosphere: (a,b) MIL-101(Cr) at 30 °C (full spectrum and zoomed 1750-1350 cm^{-1} region), (c,d) MIL-101(Cr)-MEL at 30 °C, and (e,f) MIL-101(Cr)-MEL at 60 °C.

Table S1. Amine functionalities and isosteric heats of CO_2 adsorption at zero coverage used in Figure 12.

Adsorbent	Amine functionality	$-\Delta H_{ads}$ (kJ mol^{-1})	Ref.
MIL-101(Cr)	Pristine framework	24	This work
MIL-101(Cr)-MEL	Melamine	37	This work
PM24@MIL-101(Cr)	Polymethacrylamide (PM24)	50	[1]
PVAm(0.7)@MIL-101	Polyvinylamine (PVAm)	50	[2]
MIL-101(Cr)-NH_2	Aminoterephthalate linker (−NH_2)	55	[3]
M101-PDA(0.6)	1,3-Propanediamine (PDA)	54	[4]
MIL-101(Cr)-TEPA(30)	Tetraethylenepentamine (TEPA)	62	[5]
M101-BDA(0.6)	1,4-Diaminobutane (BDA)	67	[4]
EN-MIL-100(Cr)	Ethylenediamine (EN)	80	[6]
MMEN-MIL-100(Cr)	N,N′-Dimethylethylenediamine (MMEN)	80	[6]
Cr-MIL-101-SO_3-TAEA	Tris(2-aminoethyl)amine (TAEA)	87	[7]
TAEA@MIL-101(Cr)	Tris(2-aminoethyl)amine (TAEA)	119	[8]

References

(1) Yoo, D. K.; Yoon, T. U.; Bae, Y. S.; Jhung, S. H. Metal-organic framework MIL-101 loaded with polymethacrylamide with or without further reduction: effective and selective CO_2 adsorption with amino or amide functionality. *Chem. Eng. J.* **2020**, *380*, 122496.

(2) Shin, S.; Yoo, D. K.; Bae, Y. S.; Jhung, S. H. Polyvinylamine-loaded metal–organic framework MIL-101 for effective and selective CO_2 adsorption under atmospheric or lower pressure. *Chem. Eng. J.* **2020**, *389*, 123429.

(3) Park, J. M.; Cha, G. Y.; Jo, D.; Cho, K. H.; Yoon, J. W.; Hwang, Y. K.; Lee, S. K.; Lee, U. H. Amine and fluorine co-functionalized MIL-101(Cr) synthesized via a mixed-ligand strategy for CO_2 capture under humid conditions. *Chem. Eng. J.* **2022**, *444*, 136476.

(4) Lee, G.; Jhung, S. H. Carbon dioxide capture using diaminoalkane-grafted MIL-101(Cr)s: Critical role of geometric configuration of loaded amines. *Sep. Purif. Technol.* **2024**, *337,* 126445.

(5) Rim, G.; Priyadarshini, P.; Song, M. G.; Wang, Y.; Bai, A.; Realff, M. J.; Lively, R. P.; Jones, C. W. Support Pore Structure and Composition Strongly Influence the Direct Air Capture of CO_2 on Supported Amines. *J. Am. Chem. Soc.* **2023**, *145* (13), 7190-7204.

(6) Cabello, C. P.; Berlier, G.; Magnacca, G.; Rumori, P.; Palomino, G. T. Enhanced CO_2 adsorption capacity of amine-functionalized MIL-100(Cr) metal-organic frameworks. *CrystEngComm* **2015**, *17* (2), 430-437.

(7) Li, H.; Wang, K.; Feng, D.; Chen, Y. P.; Verdegaal, W.; Zhou, H. C. Incorporation of Alkylamine into Metal–Organic Frameworks through a Brønsted Acid–Base Reaction for CO_2 Capture. *ChemSusChem* **2016**, *9* (19), 2832–2840.

(8) Zhong, R.; Yu, X.; Meng, W.; Liu, J.; Zhi, C.; Zou, R. Amine-Grafted MIL-101(Cr) via Double-Solvent Incorporation for Synergistic Enhancement of CO_2 Uptake and Selectivity. *ACS Sustain. Chem. Eng.* **2018**, *6* (12), 16493-16502.
